# Robot-Assisted Urachal Excision and Partial Cystectomy for Urachal Pathologies: Systematic Review with Insights from Single-Center Experience

**DOI:** 10.3390/jcm14041273

**Published:** 2025-02-14

**Authors:** Rafał B. Drobot, Grzegorz Stawarz, Marcin Lipa, Artur A. Antoniewicz

**Affiliations:** 1 Urology Department, Institute of Medical Sciences, Faculty of Medicine, Collegium Medicum, Cardinal Stefan Wyszyński University in Warsaw, Bursztynowa St. 2, 04-479 Warsaw, Poland; m.lipa@uksw.edu.pl (M.L.); a.antoniewicz@uksw.edu.pl (A.A.A.); 2 Department of Urology and Urological Oncology, Multidisciplinary Hospital in Warsaw-Miedzylesie, Bursztynowa St. 2, 04-479 Warsaw, Poland

**Keywords:** robot-assisted surgery, urachal pathology, partial cystectomy, urachal excision, urachal adenocarcinoma, oncological outcomes

## Abstract

**Background:** Urachal pathologies, while rare, carry a risk of malignant transformation. Robot-assisted urachal excision and partial cystectomy (RAUEPC) is a minimally invasive technique that offers potential advantages, but the available evidence remains limited. This study aims to evaluate the outcomes of RAUEPC for benign and malignant urachal pathologies through a systematic review and single-center experience. **Methods:** A systematic review was conducted using PubMed, Scopus, the Cochrane Library, and ScienceDirect (last search: 1 November 2024). Inclusion criteria encompassed studies reporting on RAUEPC for urachal pathologies, while non-robotic approaches and incomplete data were excluded. Risk of bias was assessed using the Newcastle-Ottawa Scale for cohort studies and the JBI Critical Appraisal Checklist for Case Reports. Descriptive statistics summarized continuous data (means, medians, 95% confidence intervals), and chi-square tests analyzed associations between categorical variables. Heterogeneity analysis was infeasible, necessitating narrative synthesis. Institutional retrospective data from three cases (2021–2024) were included for comparison. This study was registered in PROSPERO (CRD42024597785). No external funding was received. **Results:** A total of 44 studies (n = 145) met the inclusion criteria. Benign lesions accounted for 66.2% and malignant lesions for 33.8%. Mean operative time was 177.8 min (cumulative), 162.7 min (benign), 192.2 min (malignant), 85.33 min (institutional, 95% CI: 74.13–96.53). Mean blood loss was 85.4 mL (cumulative), 99.5 mL (benign), 72.7 mL (malignant), 216.66 mL (institutional). Mean hospital stay was 3.64 days (cumulative), 3.26 days (benign), 4.36 days (malignant), 6.33 days (institutional, 95% CI: 3.46–9.20). Complications occurred in 10.04% (cumulative), 11.82% (benign), 8.57% (malignant), with one minor event (Clavien–Dindo II) in institutional cases. No conversions to open surgery were reported. All cases achieved complete excision with no R1 resections. No recurrences were observed at 10.66-month (institutional) mean follow-up. **Conclusions:** RAUEPC appears to be a feasible and safe approach with promising short-term outcomes. The associations between symptoms and diagnostic methods highlight its utility. The limitations of the evidence include small sample sizes and retrospective designs. Further prospective studies are needed to validate these findings.

## 1. Introduction

### 1.1. Epidemiology of Urachal Pathology and Clinical Manifestation

Although urachal anomalies are infrequent, they pose significant clinical challenges owing to their potential for malignant transformation. The reported incidence of urachal anomalies in the literature varies, ranging from 1 in 5000 to 1 in 3 adults with asymptomatic, clinically insignificant remnants at autopsy [1,2,3,4,5,6]. Most asymptomatic urachal remnants are incidentally discovered. However, symptomatic cases requiring definitive surgical intervention frequently present with hematuria (49%) and abdominal pain (27%) [1,7]. In severe cases, infected urachal cysts can present with umbilical discharge, periumbilical pain, and peritonitis [8]. Pediatric presentations often include urinary symptoms such as dysuria and suprapubic pain [9], although rare presentations in adults, such as urachal cysts causing dyspareunia and dysorgasmia, have also been reported [7].

### 1.2. Embryogenesis of the Urachus and Risk of Malignant Transformation

**Figure 1:**
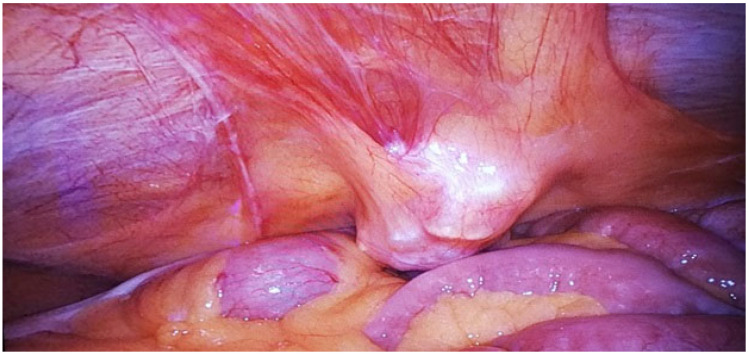
Urachal adenocarcinoma.

During fetal development, the urachus is a tubular structure connecting the dome of the bladder to the umbilicus, originating from the cloaca and allantois [10]. Normally, the urachus is obliterated before birth, forming the median umbilical ligament. Failure of this obliteration can result in various urachal anomalies, including a patent urachus (complete failure of obliteration, resulting in a continuous channel between the bladder and umbilicus), urachal sinus (partial obliteration, leaving an open channel at the umbilical end), urachal cyst (a fluid-filled cavity forming in the midportion due to closure at both ends), and vesicourachal diverticulum (an outpouching connected to the bladder) [1]. These anomalies can lead to clinical complications, such as infections and, in rare cases, malignant transformation into urachal adenocarcinoma [10]. Certain factors may increase this risk, including chronic inflammation and recurrent infections, which predispose urachal remnants to malignant changes due to prolonged irritation of epithelial-lined structures [11]. The risk of malignancy is higher in persistent anomalies, especially urachal cysts, which account for up to 54% of urachal anomalies and are prone to inflammation [12]. Urachal adenocarcinoma (Figure 1) comprises < 1% of all bladder cancers, predominantly affecting men over the age of 50 [10,13].

### 1.3. Staging of Urachal Cancer

Understanding the progression of urachal carcinoma is crucial for effective treatment planning, which is facilitated by staging systems such as the Sheldon staging system. The Sheldon staging system is commonly used for urachal adenocarcinoma due to the unique nature of this malignancy (Table 1) [14]. It is frequently favored over the TNM classification for urachal carcinoma because of its specificity in addressing the unique patterns of local invasion, including stages that detail tumor extension to the abdominal wall, peritoneum, and other adjacent structures. Additionally, the Mayo staging system is commonly used, as it provides a simplified approach with potential superior prognostic value, particularly in multivariate analyses. This makes it a valuable alternative for stratifying risk and guiding treatment decisions in clinical practice [15].

Given the rarity and aggressive nature of urachal adenocarcinoma, early detection, and accurate staging are essential for effective management.

### 1.4. Diagnostic Imaging

Ultrasound is typically the first imaging modality used for evaluating suspected urachal anomalies due to its accessibility and non-invasive nature, with diagnostic success rates between 75% and 100% for detecting urachal cysts [9]. This variability may be influenced by factors such as operator expertise, patient anatomy (e.g., body habitus or cyst location), and the quality of ultrasound equipment, all of which affect imaging accuracy. For complex cases or those with inconclusive ultrasound findings, computed tomography (CT) and magnetic resonance imaging (MRI) can provide additional detailed information. CT is beneficial for assessing the extent of the disease and identifying characteristic features, such as calcifications and mucinous content. Calcifications, which are present in 50–70% of cases, are pathognomonic of urachal adenocarcinomas. MRI provides superior soft-tissue contrast, which can help evaluate the local extent of the tumor and its relationship with adjacent structures [7]. A combination of imaging modalities is often necessary to provide comprehensive urachal pathology assessment, which in turn guides diagnosis and treatment planning.

### 1.5. Treatment Modalities, Current Approaches, and Challenges in Management

The treatment of urachal anomalies depends on the clinical presentation and symptom severity. Infected urachal cysts may initially be managed with antibiotics, although definitive treatment often requires surgical excision to prevent recurrence and complications, such as sepsis or malignant transformation [16]. Despite advancements in surgical techniques, the management of urachal anomalies remains challenging due to their rarity and oncogenic potential, underscoring the critical need for effective interventions to mitigate symptoms and prevent malignant transformation. Similar to the variability observed in the guidelines for UTUC, the management of urachal pathologies suffers from a lack of standardized approaches [17]. The current literature lacks comprehensive, evidence-based guidelines for urachal anomalies, emphasizing the need for more structured treatment protocols. The management of urachal adenocarcinoma remains particularly controversial due to its rarity and the absence of consensus on optimal treatment strategies. Further prospective studies and consensus-building efforts are essential to refine diagnostic and therapeutic strategies for these rare conditions. Historically, radical cystectomy has been the standard treatment. However, bladder-sparing approaches have shown promising outcomes, such as partial cystectomy with en-bloc resection of the urachus and umbilicus [18,19]. Although open surgical approaches have traditionally been the standard, laparoscopic and robot-assisted techniques are increasingly being adopted. Robot-assisted surgery represents a significant advancement in minimally invasive techniques and is increasingly applied across various fields of urology. It combines precision with enhanced visualization, providing surgeons greater control over complex anatomical areas. In the context of urachal surgery, robot-assisted techniques facilitate precise excision with minimal trauma, which is particularly advantageous due to the anatomical location of urachal anomalies. The robot-assisted laparoscopic approach has emerged as a feasible and safe method, offering minimal complications, quicker recovery, and reduced surgical morbidity and postoperative pain while maintaining oncologic control [18,19,20,21]. Although robotic surgery offers clear benefits, challenges such as high costs, limited availability, and a steep learning curve for surgeons also exist. Addressing these factors is essential for developing a balanced perspective on the widespread adoption of robot-assisted techniques. Due to the rare occurrence of urachal adenocarcinoma, therapeutic options are still being explored. As shown in Table 2, ongoing clinical trials are investigating novel interventions, including chemotherapy, immunotherapy, and targeted therapies. Despite these ongoing studies, further research is needed to establish optimal treatment protocols and improve clinical outcomes in patients with this rare malignancy.

### 1.6. Novelty and Contribution of This Study

**Figure 2:**
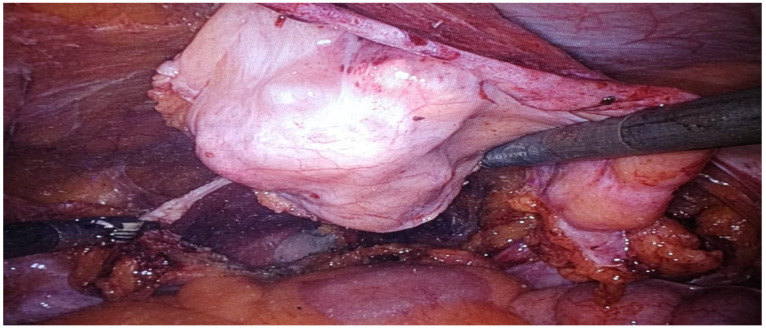
Robot-assisted urachal excision (urachal pathology separated from the posterior sheath of rectus abdominis muscle).

**Figure 3:**
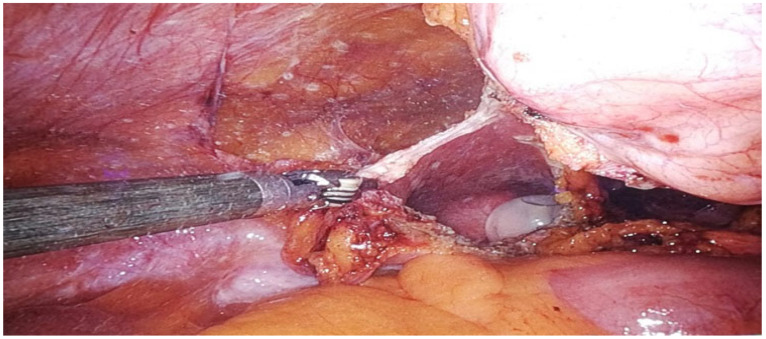
Robot-assisted partial cystectomy (en bloc resection of the urachal pathology along with bladder cuff).

This study represents a significant contribution to the limited body of research on robot-assisted urachal surgery, offering the first systematic review focused explicitly on robot-assisted urachal excision (Figure 2) and partial cystectomy (Figure 3). Insights derived from single-center experience provide a detailed examination of the learning curve associated with adopting robotic techniques in urachal surgery. This critical factor may influence clinical outcomes and inform future practice. Furthermore, this research enhances the existing knowledge base by evaluating long-term outcomes and advocating for standardized protocols in patient selection and surgical techniques. By addressing these dimensions, this study aims to establish a foundation for evidence-based guidelines that can enhance consistency in managing urachal anomalies through robotic approaches.

To the best of our knowledge, our case series represents a pioneering contribution to Polish medical literature, documenting the use of robot-assisted surgery for urachal pathology and emphasizing advancements in minimally invasive urological surgery within the country.

## 2. Aim

The principal review question was to examine the feasibility, safety, complication rates, and both short- and long-term outcomes of robot-assisted urachal excision and partial cystectomy (RAUEPC) for treating benign and malignant urachal pathologies, drawing on evidence from both the existing literature and institutional experience.

In this context, the primary objectives focus on assessing the feasibility (including surgical time, intraoperative blood loss, and conversion rate) of RAUEPC, as well as evaluating the efficacy (including complete excision rate or margin status) and safety (including intra- and postoperative complications, hospital stay duration and readmission rates) of the procedure.

The secondary objectives involve analyzing short-term clinical outcomes (including umbilical removal and lymphadenectomy, lymph node involvement or metastasis, and histopathological findings), as well as long-term follow-up outcomes (oncological results, including recurrence rate and need for adjuvant therapy) and identifying factors that influence diagnostic accuracy, surgical success, and patient outcomes.

By juxtaposing institutional outcomes with a thorough review of the extant literature, this study aimed to appraise the current standards of care, elucidate potential areas for enhancement, and articulate evidence-based recommendations for future clinical practice in the management of both benign and malignant urachal anomalies, with separate analyses of institutional outcomes, benign pathologies, malignant pathologies, and a cumulative synthesis of findings.

## 3. Material and Methods

This systematic review was conducted in accordance with the Preferred Reporting Items for Systematic Reviews and Meta-Analyses (PRISMA) 2020 statement [22]. The study protocol was registered prior to initiation in the International Prospective Register of Systematic Reviews (PROSPERO) (Registration Number: CRD42024597785, available at https://www.crd.york.ac.uk/PROSPERO/display_record.php?RecordID=597785 (accessed on 2 February 2025)). As the review progressed, the protocol was updated to include the JBI Critical Appraisal Checklist for Case Reports as an additional tool for assessing risk of bias. This update was implemented due to the significant number of case reports identified during preliminary searches, for which the Newcastle–Ottawa Scale (NOS) was not specifically designed.

### 3.1. Search Strategy

To ensure a systematic review of the literature, a structured search strategy was employed using specific keywords and Medical Subject Headings (MeSH) terms. The last search was conducted on 1 November 2024. Automation tools were not used in the screening or selection processes. No limitations were imposed on the publication date, although the search was restricted to studies published in English with full-text availability. Boolean operators were used to effectively combine these keywords. Three review authors (RBD, ML, and GS) independently conducted a comprehensive literature search across four electronic databases (PubMed, Scopus, Cochrane Library, and ScienceDirect) using the following search string:

- (“robotic” OR “robot-assisted” OR “robot-assisted laparoscopic”) AND (“urachal” OR “urachus” OR “urachal anomalies” OR “urachal cyst” OR “urachal diverticulum” OR “urachal adenoma” OR “urachal adenocarcinoma”) AND (“removal” OR “excision” OR “partial cystectomy”)

Due to the limitation of Boolean operators in the ScienceDirect database search engine, which supports a maximum of eight operators, the above search string was divided into the following three separate queries for this database:

- (“robotic” OR “robot-assisted” OR “robot-assisted laparoscopic”) AND (“urachal” OR “urachus”) AND (“removal” OR “excision” OR “partial cystectomy”)
- (“robotic” OR “robot-assisted” OR “robot-assisted laparoscopic”) AND (“urachal anomalies” OR “urachal cyst”) AND (“removal” OR “excision” OR “partial cystectomy”)
- (“robotic” OR “robot-assisted” OR “robot-assisted laparoscopic”) AND (“urachal diverticulum” OR “urachal adenoma” OR “urachal adenocarcinoma”) AND (“removal” OR “excision” OR “partial cystectomy”)

### 3.2. Inclusion and Exclusion Criteria

The eligibility of retrieved studies was evaluated using the population, intervention, comparison, outcome(s), and study design (PICOS) framework. The inclusion criteria for this systematic review were as follows:

(P)opulation: Patients with urachal pathologies (both benign and malignant)

(I)ntervention: RAUEPC

(C)omparison: Institutional data of patients who underwent RAUEPC

(O)utcomes: The collected data include the following:

- Reason for Surgery
- Symptoms
- Imaging Method
- Cystoscopy and/or preoperative TURBT results
- Staging
- Umbilicus Removal
- Lymphadenectomy
- Complications
- Hospital Stay
- Histopathological Findings
- Robotic System
- Total Operation and Console Time
- Blood Loss
- Patient Characteristics
- Follow-Up
- Adjuvant Therapy

(S)tudy Design: Retrospective cohort studies, case series, case reports, and other observational studies or reports where detailed surgical, clinical, outcome, and follow-up data are available were included.

The exclusion criteria for this systematic review included studies involving animal models or in vitro research, case reports lacking comprehensive surgical or outcome data, and studies employing non-robotic surgical techniques.

Additionally, this study retrospectively analyzed all patients who underwent RAUEPC at the Urology Department, Institute of Medical Sciences, Faculty of Medicine, Collegium Medicum, Cardinal Stefan Wyszyński University in Warsaw. The inclusion criteria were symptomatic patients hospitalized between 2021 and 2024 with imaging-confirmed or suspected urachal tumors requiring surgical intervention. Patients with non-urachal bladder tumors, other malignancies, or contraindications to robotic surgery were excluded from the study.

### 3.3. Screening Process and Data Extraction

Three reviewers (RBD, ML, and GS) manually screened the full-text articles for inclusion in the systematic review, following pre-specified eligibility criteria. Each reviewer independently assessed the titles, abstracts, and full texts to determine eligibility. The reviewers were blinded to each other’s decisions during the initial screening to minimize bias. Any disagreements were resolved through discussion, and if necessary, the senior co-investigator (AAA) was consulted for final decisions.

Data were manually extracted by three reviewers (RBD, ML, and GS) using a standardized data extraction template. The extracted data included study design and methodology, participant demographics and baseline characteristics, key outcomes (e.g., operative time, blood loss, complications), surgical techniques, and follow-up details. Discrepancies between reviewers were resolved through discussion among the three reviewers, with final decisions made in consultation with the senior co-investigator (AAA) if necessary. In cases of missing or unclear data, the authors were contacted to obtain additional information. If missing data could not be retrieved, they were documented, and this limitation was noted in the review.

Institutional data were extracted from retrospective patient medical records using the same standardized data extraction template, ensuring compliance with the General Data Protection Regulation (GDPR) to safeguard personal data privacy.

### 3.4. Quality Assessment and Risk of Bias (RoB)

The Newcastle–Ottawa Scale (NOS) was used to assess the RoB and quality of cohort studies, case–control studies, and case series, focusing on selection, comparability, and outcome assessment [23]. Each study was independently reviewed by three assessors (RBD, ML, and GS), with discrepancies resolved through discussion or by involving a senior reviewer (AAA). Studies scoring below a predetermined threshold of 6 points were excluded to ensure that only high-quality evidence was incorporated into the final analysis. This approach aligns with the Agency for Healthcare Research and Quality (AHRQ) guidelines, which suggest that studies with 3 or 4 stars in the selection domain, 1 or 2 stars in the comparability domain, and 2 or 3 stars in the outcome/exposure domain can be considered of good quality.

For case reports, the JBI Critical Appraisal Checklist was used to evaluate the methodological quality and the extent to which each study addressed potential biases in its design, conduct, and analysis [24]. Similarly, each case report was independently reviewed by three assessors (RBD, ML, and GS), and any discrepancies were resolved through discussion or senior reviewer input (AAA). This quality assessment framework ensures that the conclusions of the review are based on robust and reliable data, thereby enhancing the credibility and applicability of the findings to clinical practice.

### 3.5. Heterogeneity Tests

The Cochran’s *Q* and Higgins’ *I*^2^ statistics for heterogeneity, as outlined in the PROSPERO protocol, were not conducted due to the absence of key variables required for the analysis. These missing variables include effect sizes or measures of interest (e.g., odds ratios, risk ratios, proportions, or means), associated standard errors or variances, sample sizes, study labels or subgroup identifiers (optional, to differentiate studies or subgroups), weighting factors (inversely proportional to variance), confidence intervals (lower and upper bounds), event counts for binary outcomes (treatment and control groups), group sizes for binary outcomes (total participants in treatment and control groups), subgroup identifiers (to compare predefined groups such as countries or sexes), and outcome measure types (continuous, binary, or time-to-event).

### 3.6. Statistical Analysis and Data Synthesis

All statistical analyses were performed using Statistica 13.3 (StatSoft Inc., Tulsa, OK, USA). Data synthesis was conducted if at least five studies provided comparable data for a given outcome. Descriptive statistics were used to summarize the data. For continuous quantitative variables (e.g., hospital stay, operative time, console time, estimated blood loss, and age), we calculated arithmetic means, medians, minimum, and maximum values, along with 95% confidence intervals to present the central tendency. For any study that reported a range for continuous clinical variables, such as blood loss (mL) or operation time (min), the mid-point of the range was calculated prior to performing the descriptive analysis. For categorical qualitative variables (e.g., symptoms, imaging method used, umbilicus removal, lymphadenectomy, peri- and postoperative complications, and pathological outcomes), we reported frequencies and percentages to illustrate the distribution of these events. In cases where data were missing, we employed pairwise deletion, analyzing the available data for each specific test or summary measure. This approach was selected to maximize data retention while acknowledging any limitations in the overall sample size due to incomplete records. If missing data exceeded 20% for any outcome, sensitivity analyses were performed to evaluate the potential impact on the results. Prior to conducting the chi-square tests, assumptions were checked to ensure statistical validity. These included: (1) independence of observations, which was met by the study design; (2) adequate expected cell counts, with each cell in cross-tabulations expected to have at least five cases to maintain the reliability of the chi-square approximation; and (3) homogeneity of sample proportions across groups for comparisons. The level of statistical significance for all analyses was set at *p* < 0.05. All *p*-values were reported exactly, except when they were below the threshold of significance, in which case they were denoted as *p* < 0.05. Finally, we evaluated the certainty of evidence for each outcome using the GRADE approach, assessing factors such as the risk of bias, consistency, directness, precision, and publication bias to gauge confidence in the results.

### 3.7. Ethical Considerations

The study was conducted in accordance with the ethical standards set by both the institutional and national research committees, as well as the 1964 Helsinki Declaration and its subsequent amendments. Informed consent was obtained from all participants, and patient confidentiality was maintained in accordance with relevant guidelines.

## 4. Results

### 4.1. Search Results

**Figure 4:**
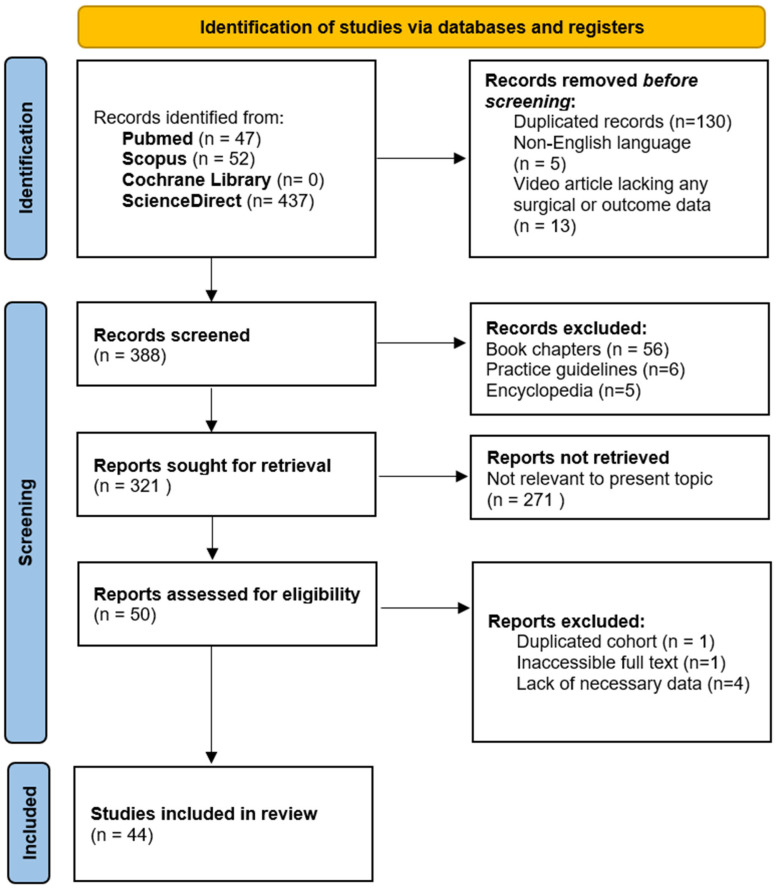
Diagram illustrating the study selection process following PRISMA 2020 guidelines.

Figure 4 provides a comprehensive overview of the study selection process. The initial systematic literature search yielded 536 publications from the PubMed, Scopus, Cochrane Library, and ScienceDirect databases. After the removal of 130 duplicate records, 5 non-English records, and 13 video-only records, 388 records were screened. During the title and abstract screening, 56 book chapters, 6 practice guidelines, and 5 encyclopedias were excluded. Of the 321 reports considered for full-text review, 271 were excluded because they were irrelevant to the topic. Additionally, 6 full-text articles were removed due to inaccessibility (1), lack of necessary data (4), or duplicate cohorts (1). Ultimately, 44 studies were included in this review.

### 4.2. Risk of Bias Assessment and Quality Evaluation

**Figure 5:**
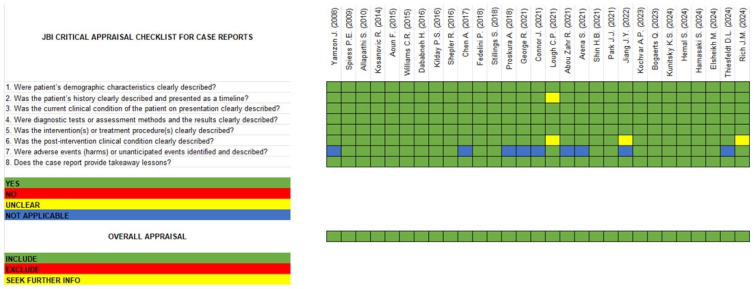
JBI Critical Appraisal Checklist for Case Reports included in systematic review [1,7,10,13,14,39,40,41,42,43,44,45,46,47,48,49,50,51,52,53,54,55,56,57,58,59,60,61,62].

Despite the retrospective design of the included studies, they demonstrated a low risk of bias and good quality across all evaluated domains, with only minor exceptions. However, potential reporting biases should also be considered. The synthesis was constrained by the absence of standardized reporting across studies, and unpublished negative findings could not be assessed, which may have introduced bias. Moreover, the reliance on retrospective studies increases the likelihood of incomplete data capture, as not all outcomes or adverse events may have been consistently reported. Efforts to address these limitations included systematic evaluation of available reports and documentation of missing outcomes. The consensus on quality ratings and RoB assessment is provided in Table 3 and Figure 5.

**Table 3:** Newcastle–Ottawa Scale scores for cohort studies, case–control studies, and case series included in the systematic review [11,25,26,27,28,29,30,31,32,33,34,35,36,37,38].

Study	Selection	Comparability	Outcome	Total
Madeb R. (2006) [25]	3	1	2	6
Nayyar R. (2009) [26]	3	1	2	6
Correa J.J. (2009) [27]	3	1	2	6
Kim D.K. (2010) [28]	3	1	2	6
Lee H.E. (2010) [29]	3	1	2	6
Tadtayev S. (2011) [30]	3	1	2	6
Raynor M. (2011) [31]	3	1	2	6
Rivera M. (2015) [32]	3	1	3	7
James K. (2015) [33]	3	1	3	7
Fode M. (2016) [34]	3	1	3	7
Ahmed H. (2017) [35]	3	1	3	7
Yong J. (2020) [36]	3	1	2	6
Osumah T.S. (2021) [37]	3	1	2	6
Perez D. (2022) [11]	3	1	2	6
Stokkel L.E. (2022) [38]	3	1	3	7

### 4.3. Overview

In this study, outcomes were first contextualized by comparing findings from the existing literature on RAUEPC for urachal pathologies, as summarized in Table 4, with institutional data. This comparative analysis positioned our results within the broader context of previously published studies. A detailed examination of the procedures performed at our institution is presented, including a breakdown of operative outcomes, complication rates, and patient recovery rates, as summarized in Table 5. This approach was employed to provide a nuanced understanding of both external evidence and institutional experience, highlighting similarities and differences in clinical outcomes.

#### 4.3.1. Sex and Age Distribution

**Figure 6:**
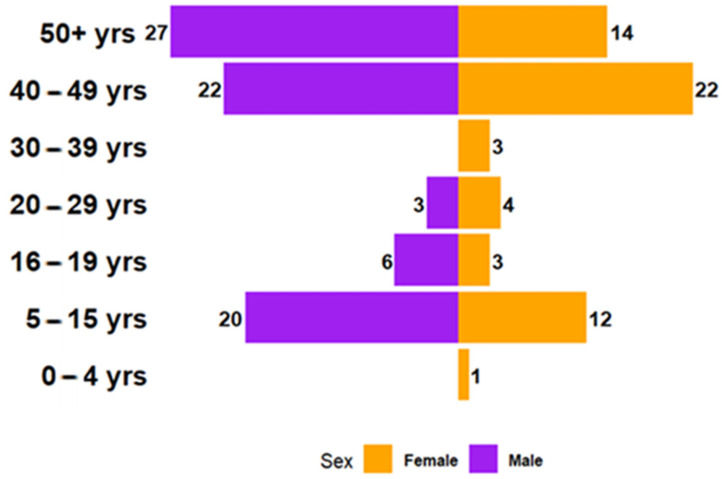
Sex distribution by age group in patients undergoing RAUEPC for benign and malignant urachal pathologies in the included studies.

Among the total cohort of 145 patients (Figure 6), females accounted for 60 cases (GR: 0.71), while males made up 85 cases (GR: 1.42). In the youngest age group (0–4 years), only one female patient (0.73%) was recorded. The 5–15 years category saw a notable increase in cases, with 14.6% male and 8.76% female. In the 16–19 years group, males (4.38%) slightly outnumbered females (2.19%). Among young adults (20–29 years), females (2.92%) slightly surpassed males (2.19%). In older age groups, the sex distribution became more balanced or male-dominated. For instance, in the 40–49 years group, males and females each accounted for 16.06% of the cases. However, in the 50+ years category, males (19.71%) significantly outnumbered females (10.22%), indicating a strong male predominance in older patients.

In our cohort, all three patients were male, consistent with the observed male predominance reported in the literature, particularly in older age groups. The patients’ ages ranged from 44 to 66 years, with a mean age of 52.66 years, aligning with the typical age distribution for urachal pathologies.

#### 4.3.2. Reasons for Surgery

**Figure 7:**
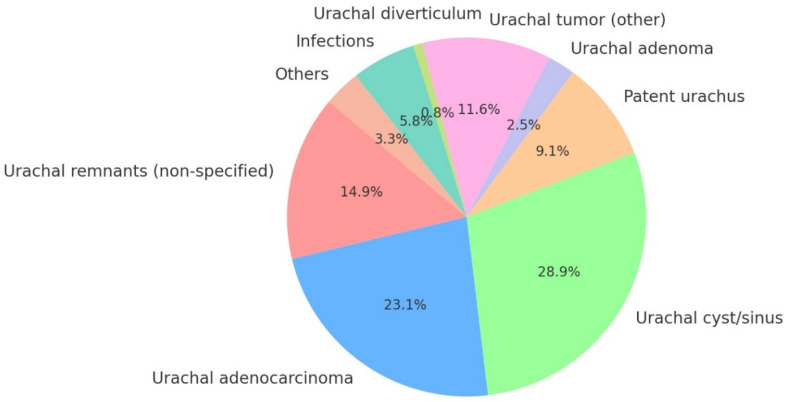
Reasons for RAUEPC for benign and malignant urachal pathologies in the included studies.

Among the reasons for surgery (Figure 7), urachal cyst/sinus (35 [28.92%]) was the most prevalent. Malignant conditions (based on preoperative biopsy/TURBT results only), such as urachal adenocarcinoma (28 [23.14%]), also played a notable role, emphasizing the diverse pathology of urachal abnormalities. Intermediate prevalence was noted for non-specified urachal remnants (18 [14.87%]), other urachal tumors (14 [11.57%]), and patent urachus (11 [9.09%]). Infections constituted a rare cause for surgery (7 [5.78%]).

In our case series, the primary indications for surgery were two urachal tumors suspected on CT, and one adenocarcinoma confirmed by preoperative TURBT.

#### 4.3.3. Symptoms

The most prevalent symptom was hematuria (29.89%). Abdominal pain was another commonly reported symptom, observed in 18.39% of the cases. Dysuria (13.79%) and UTI (9.20%) were other frequent symptoms. These data highlight the variability in symptom presentation and the importance of individualized clinical evaluations for accurate diagnosis. A summary is presented in Table 6.

Among our cases, suprapubic pain was noted in two patients (66.6%), while hematuria was present in one patient (33.3%), reflecting the symptom variability described in the literature.

#### 4.3.4. Imaging Methods

CT scans (31 [43.05%]) were the most commonly employed imaging method, followed by MRI (22 [30.55%]). Other imaging methods included ultrasound (US) (9 [12.50%]). PET scans (3 [4.16%]) were less commonly used. Rarely utilized methods included CT urography (2 [2.77%]), cystograms (1 [1.38%]), voiding cystourethrogram (VCUG) (1 [1.38%]), and others (1 [1.38%]).

All cases (100%) in our series were evaluated using CT imaging, reflecting its predominant role in diagnosing urachal pathologies.

#### 4.3.5. Cystoscopy Results

The most common result noted among preoperative cystoscopy descriptions was a bladder dome mass/finding (40.38%), followed by a normal cystoscopy result (11.54%) and urachal remnant ducts (9.62%). Less common findings included bladder wall thickening (3.85%) and several other findings (Table 7).

Preoperative cystoscopy in our series revealed a bladder dome mass in two cases (66.6%), while one case (33.3%) showed a normal result.

### 4.4. Primary Outcomes: Feasibility

#### 4.4.1. Data from Institutional Results

Institutional data showed a mean operative time of 85.33 min (95% CI: 74.13–96.53 min) and a mean estimated blood loss of 216.66 mL. Notably, no cases required conversion to open surgery.

#### 4.4.2. Data from a Systematic Review on Benign Urachal Pathologies

Data from a systematic review on benign urachal pathologies showed a mean operative time of 162.7 min (95% CI: 119.89–205.51 min) and a mean estimated blood loss of 99.5 mL (95% CI: 42.42–156.58 mL). Importantly, no conversions to open surgery were reported in the literature.

#### 4.4.3. Data from a Systematic Review on Malignant Urachal Pathologies

The analysis of malignant urachal pathologies revealed a mean operative time of 192.2 min (95% CI: 149.84–234.52 min) and an estimated blood loss averaging 72.7 mL (95% CI: 42.88–102.55 mL). As in the benign cases, no conversions to open surgery were documented.

#### 4.4.4. Cumulative Data from a Systematic Review on Benign and Malignant Urachal Pathologies

A combined analysis of benign and malignant urachal pathologies showed a mean operative time of 177.8 min (95% CI: 147.1–208.4 min) with a median of 150 min, ranging from 75 to 327 min (CV = 0.42). Reported blood loss in the literature averaged 85.4 mL (95% CI: 52.6–118.1 mL) with a median of 75 mL, ranging from 5 to 203 mL (CV = 0.72).

### 4.5. Primary Outcomes: Efficacy

#### 4.5.1. Data from Institutional Results

All three institutional cases underwent complete excision. Margin status was not documented for the two benign cases, while the malignant case achieved R0 resection.

#### 4.5.2. Data from a Systematic Review on Benign Urachal Pathologies

Margin status and resection completeness were not reported for benign cases in the systematic review.

#### 4.5.3. Data from a Systematic Review on Malignant Urachal Pathologies

All malignant cases underwent complete resection, with no reports of positive surgical margins.

#### 4.5.4. Cumulative Data from a Systematic Review on Benign and Malignant Urachal Pathologies

Complete excision was achieved in all cases included in the analysis, with no instances of R1 resection.

### 4.6. Primary Outcomes: Safety

#### 4.6.1. Data from Institutional Results

Institutional data reported one minor complication (Clavien–Dindo Grade II: red blood cell transfusion), with no major adverse events. The mean hospital stay was 6.33 days (95% CI: 3.46–9.20). No readmissions were observed in our cohort.

#### 4.6.2. Data from a Systematic Review on Benign Urachal Pathologies

The majority of patients (88.18%) did not experience any complications. Minor complications (Grade I and II) were observed in 3.64% of cases, including urinary tract infections requiring antibiotics (1.82%) and persistent abdominal pain (0.91%). Major complications (Grade IIIA, IIIB, and IVa) occurred in 8.18% of cases. The most frequent was fascial rupture (2.73%). Other significant complications included bladder leakage (0.91%), small bowel perforation requiring surgical repair (0.91%), bleeding from the spleen (0.91%), infected hematoma (0.91%), postoperative abscess requiring reoperation (0.91%), and urosepsis (0.91%). For benign urachal pathologies, the mean hospital stay was 3.26 days (95% CI: 2.03–4.48 days, CV = 0.95), indicating relatively high variability in hospitalization duration. No readmissions were reported.

#### 4.6.3. Data from a Systematic Review on Malignant Urachal Pathologies

The vast majority of patients (91.43%) had an uneventful postoperative course without complications. Minor complications (Grade I and II) were not observed in this cohort. However, major complications (Grade IIIA, IIIB, and V) occurred in 8.57% of cases. The most severe was an arterial occlusion (2.86%), classified as Grade V, which resulted in patient mortality. Other significant complications included bowel obstruction requiring surgical resection and readmission (2.86%) and urinary leakage necessitating further intervention (2.86%). In malignant cases, the mean hospital stay was 4.36 days (95% CI: 2.92–5.80 days, CV = 0.80), showing somewhat lower variability compared to benign cases. One patient required readmission.

#### 4.6.4. Cumulative Data from a Systematic Review on Benign and Malignant Urachal Pathologies

As summarized in Table 8, most patients (88.96%, 129 cases) experienced no complications. While 2.76% of patients experienced minor complications (Grade I and II; 4 cases), major complications (Grade IIIA, IIIB, IVa, and V) were observed in 8.28% of cases (12 cases). Among these, fascia rupture (3 [2.07%]) was the most frequent.

Across all cases, the mean hospital stay was 3.64 days (95% CI: 2.70–4.59 days, CV = 0.86), demonstrating considerable variation in the length of hospitalization among patients. In 2006, the longest average stay of 22 days was recorded, although based on a single case. From 2009 to 2024, the average stay generally declined, with notable exceptions in 2022 (7.33 days based on three cases) and 2021 (4.75 days based on eight cases). The shortest average stay occurred in 2023, with 1 day across two cases. The increased sample sizes in 2021 and 2024 (eight and six cases, respectively) provide more robust averages compared to earlier years. This suggests an overall trend toward shorter hospital stays over time.

A single readmission was recorded, accounting for 0.69% of the 145 cases reviewed.

### 4.7. Secondary Outcomes: Short-Term Clinical Outcomes

#### 4.7.1. Data from Institutional Results

Lymphadenectomy and umbilicus removal were not performed in our cohort, as they were not indicated based on preoperative staging. Postoperative histopathological analysis identified benign lesions in two cases and mucinous cystadenocarcinoma in one case, a distribution consistent with the existing literature. Since no lymph node involvement or metastatic disease was detected in any case, lymphadenectomy was deemed unnecessary.

#### 4.7.2. Data from a Systematic Review on Benign Urachal Pathologies

Umbilicus removal was performed in 71.82% of cases, reflecting its routine surgical application, whereas lymphadenectomy was conducted in only 8.18% of cases, with reporting unavailable in 10.91%. Given the benign nature of the lesions in this subgroup, it is unsurprising that no cases demonstrated lymph node involvement or distant metastasis, further confirming their non-aggressive behavior. Within the benign subgroup, urachal remnants were the most frequently identified pathology, constituting 31.03% (45/145) of all cases and 46.88% (45/96) of all benign findings. Similarly, urachal cysts were observed in 23.45% (34/145) of the entire cohort and represented 35.42% (34/96) of benign lesions, further highlighting their clinical prevalence. Less common benign entities included patent urachus (4 cases, 2.76% of the total; 4.17% of benign lesions), and diverticulum, leiomyoma, cystadenoma, and villous adenoma, each contributing to 1.38% (2/145) of total cases and 2.08% of benign pathologies. Rare benign conditions, such as fibrovascular necrotizing granulomatous tissue, hamartoma, and myofibroblastic tumor, accounted for only 0.69% (1/145) each, underscoring their limited clinical incidence.

#### 4.7.3. Data from a Systematic Review on Malignant Urachal Pathologies

Umbilicus removal was performed in 97.14% of cases, underscoring its routine surgical application, whereas lymphadenectomy was conducted in 68.57% of cases, indicating a more selective approach. Distant metastases were observed in 4.08% (2/49) of malignant cases, while lymph node involvement was present in 16.33% (8/49), highlighting the potential for regional and systemic spread in a subset of these tumors. Malignant urachal pathologies were predominantly represented by adenocarcinoma, which constituted 32.41% (47/145) of the total cohort and comprised 95.92% (47/49) of the malignant findings. Other malignant lesions, including cystadenocarcinoma and mucinous cystic tumors with low malignant potential, were exceedingly rare, comprising 0.69% (1/145) of the total cases and 2.04% (1/49) of malignant lesions.

#### 4.7.4. Cumulative Data from a Systematic Review on Benign and Malignant Urachal Pathologies

A striking 85.7% of patients in the literature underwent umbilicus removal. Finally, lymphadenectomy was performed in 33.3% of the cases, highlighting that some patients present with advanced malignant disease requiring the removal of lymph nodes for staging or to reduce the risk of metastasis.

Postoperative histopathological analysis of 145 cases demonstrated a clear predominance of benign lesions, which accounted for 66.21% (96/145) of the cases, compared to malignant lesions, which accounted for 33.79% (49/145).

Of the total lymphadenectomy cases, 83.33% showed no lymph node involvement, while 16.67% had lymph node involvement. An even more pronounced difference was observed in metastasis rates, where 95.23% did not present metastases, while only 4.77% showed metastatic disease.

### 4.8. Secondary Outcomes: Long-Term Follow-Up Outcomes

#### 4.8.1. Data from Institutional Results

Institutional follow-up showed no recurrences (0%) and a mean duration of 10.66 months (95% CI: 11.74–33.07). In our series, no patients required adjuvant therapy, reflecting the absence of advanced or recurrent disease.

#### 4.8.2. Data from a Systematic Review on Benign Urachal Pathologies

An assessment of the included studies demonstrated an average follow-up period of 9.90 months, with a median of 6.00 months (95% confidence interval: 3.60–16.20), and a coefficient of variation of 1.05, indicating substantial variability. As these studies primarily focused on benign conditions, oncological outcomes such as recurrence rates and the need for adjuvant therapy were not applicable and, consequently, not reported.

#### 4.8.3. Data from a Systematic Review on Malignant Urachal Pathologies

Analysis of the included studies revealed an average follow-up period of 10.67 months, with a median of 6.00 months (95% confidence interval: 4.19–17.14), and a coefficient of variation of 0.96, indicating high variability. Among 49 histologically confirmed malignant cases, the recurrence rate remained low, with one local recurrence (2.04%) and two port-site recurrences (4.08%) reported. Adjuvant therapy was administered to six patients (4.14%), with 5-fluorouracil and cisplatin being the most commonly used regimen (33.3%). Other therapies, including capecitabine with oxaliplatin (1), external radiotherapy with brachytherapy (1), and palliative chemotherapy (unspecified) (1), were each utilized in 16.66% of cases.

#### 4.8.4. Cumulative Data from a Systematic Review on Benign and Malignant Urachal Pathologies

The average follow-up period in the included studies was 10.27 months, with a median of 6.00 months (95% confidence interval: 6.31–14.23), ranging from 0.25 to 56 months, and a CV of 0.98, indicating high variability. Literature data indicate a low recurrence rate, with local recurrences (0.69%) and port-site recurrences (1.38%) occurring exclusively in patients with malignancy within an unselected population undergoing robot-assisted urachal excision and partial cystectomy for both benign and malignant indications. Adjuvant therapy was administered to 4.14% of patients who underwent RAUEPC.

### 4.9. Comparison of Institutional Outcomes with Findings from the Systematic Review

**Figure 8:**
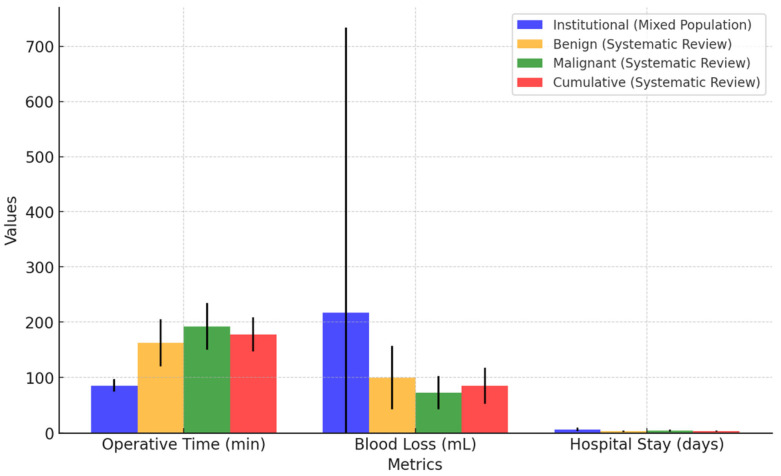
Key metrics of feasibility and safety: comparison of institutional and systematic review outcomes in RAUEPC (including 95% confidence intervals).

**Figure 9:**
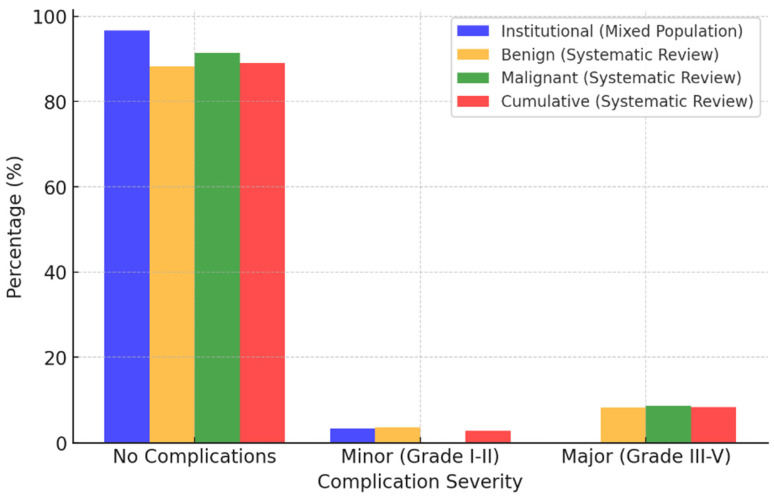
Key metrics of safety: comparison of institutional and systematic review complication rates by severity in RAUEPC (including cumulative data).

The mean operative time in institutional cases was 77.37 min shorter than in benign cases and 106.87 min shorter than in malignant cases. Estimated blood loss was 117.16 mL higher than in benign cases and 143.96 mL higher than in malignant cases. Complete resection was achieved in all institutional cases. Margin status was not documented for two benign cases, whereas systematic review data reported no instances of positive surgical margins. Institutional cases had a 5.52–5.81% lower overall complication rate. One minor complication was observed, whereas systematic review data included three additional minor complications and twelve major complications. The mean hospital stay was 3.07 days longer than for benign cases and 1.97 days longer than for malignant cases. Umbilicus removal was omitted in all institutional cases, contrasting with 71.82% of benign and 97.14% of malignant cases. Lymphadenectomy was not performed, whereas it was reported in 8.18% of benign and 68.57% of malignant cases. No recurrences were observed in institutional cases, whereas one local recurrence and two port-site recurrences were documented in the systematic review. No patients required adjuvant therapy, in contrast to six cases receiving systemic treatment in the systematic review cohort. The key findings on feasibility and safety are summarized in Figure 8 and Figure 9.

### 4.10. Factors Influencing Diagnostic Accuracy, Surgical Success and Patient Outcomes

The statistical analysis revealed a strong association between imaging methods and symptoms, as evidenced by an extremely high chi-square statistic (χ^2^ = 1761) with a *p*-value of <2.2 × 10^−16^. This result strongly suggests that the choice of imaging method is significantly influenced by the presence or type of symptoms. Patients presenting with gross hematuria were more likely to undergo advanced imaging techniques, such as CT or MRI, while those with non-specific urinary symptoms, such as frequency or urgency, were more frequently evaluated using ultrasound. A significant association was observed with adjuvant therapy (χ^2^ = 38.51, *p* = 2.42 × 10^−6^), suggesting that imaging methods may vary depending on the use of adjuvant treatment. Patients who underwent adjuvant therapy were more likely to have undergone CT imaging prior to therapy initiation. The relationship with the age group (χ^2^ = 1088.7, *p* < 2.2 × 10^−16^) highlights significant differences in imaging preferences or applications across various age categories. Specifically, younger patients (aged < 30 years) predominantly underwent ultrasound, whereas older patients (aged > 50 years) were more likely to undergo CT or MRI. Sex was strongly associated with symptoms (χ^2^ = 74.9, *p* < 0.05) and age group (χ^2^ = 27.8, *p* < 0.05), indicating differences in symptom presentation and age distribution across sexes. Males were more likely to present with gross hematuria, while females reported non-specific symptoms, such as urgency and dysuria. Sex influenced the choice of imaging method (χ^2^ = 33.6, *p* < 0.05), with males more frequently undergoing CT and females more likely to undergo ultrasound evaluations. Cystoscopy results (χ^2^ = 65.6, *p* < 0.05) revealed that males had a higher incidence of findings suggestive of malignancy than females. Sex significantly impacted the administration of adjuvant therapy (χ^2^ = 7.2, *p* < 0.05), with males slightly more likely to receive it, and reasons for surgery (χ^2^ = 97.2, *p* < 0.05), with benign conditions being more common in females and malignancies more frequent in males. A strong relationship between symptoms and age group (χ^2^ = 6727, *p* < 0.05) highlighted the significant variability in symptom presentation across different age categories. Younger patients (<30 years) more commonly presented with non-specific symptoms, such as abdominal pain and urinary frequency, while older patients (> 50 years) predominantly exhibited hematuria. The association between age group and adjuvant therapy (χ^2^ = 793.62, *p* < 0.05) revealed notable differences in therapeutic interventions among the age groups, with middle-aged patients (50–65 years) being the most likely to receive adjuvant therapy. The reason for surgery (χ^2^ = 6700, *p* < 0.05) also varied substantially, suggesting that surgical decisions are influenced by age-related factors. Younger patients often undergo surgery for benign conditions, whereas older patients are more frequently treated for malignancies. Finally, the results from the chi-square tests indicated significant associations between adjuvant therapy and both the reason for therapy (χ^2^ = 1117, *p* < 2.2 × 10^−16^) and cystoscopy results (χ^2^ = 208.7, *p* < 2.2 × 10^−16^). These findings strongly suggest a robust statistical relationship between the application of adjuvant therapy and cystoscopy outcomes, with patients with positive cystoscopy findings being more likely to receive adjuvant treatment.

**Figure 10:**
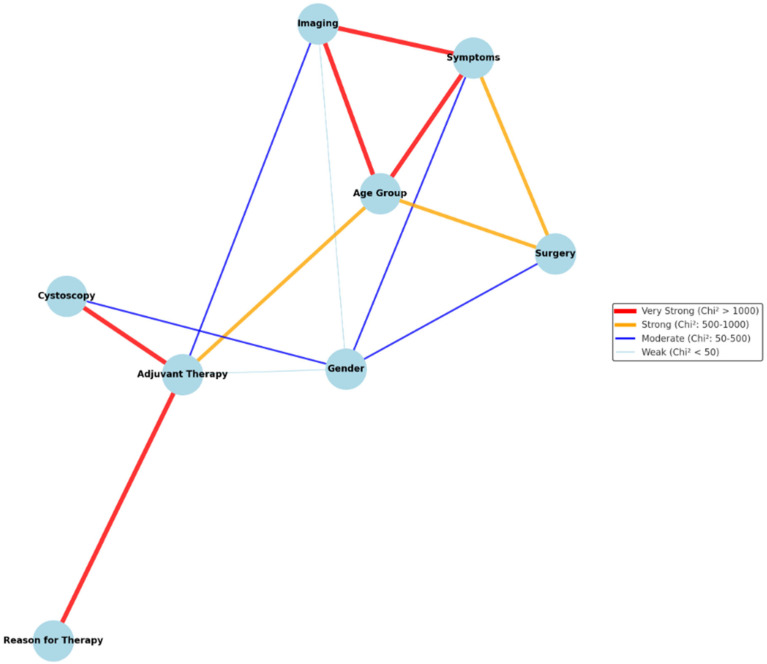
Statistically significant associations in clinical data of patients undergoing RAUEPC for benign and malignant urachal pathologies in the literature.

These associations, summarized in Figure 10, provide a comprehensive overview of statistically significant findings and evidence-based guidance (Table 9) for diagnostic and therapeutic decisions in RAUEPC for benign and malignant urachal pathologies.

## 5. Discussion

### 5.1. Advancements and Clinical Implications of Robot-Assisted Surgery in Urachal Pathologies

Robot-assisted surgery has emerged as a revolutionary method for treating urachal pathologies, providing significant advancements in surgical precision, minimal invasiveness, and improved patient recovery times. This discussion synthesizes findings from multiple studies and clinical cases.

#### 5.1.1. Precision and Control

Robotic systems offer surgeons enhanced dexterity and control, which are essential for excising urachal tumors in the anatomically confined abdominal and pelvic regions. This precision minimizes damage to surrounding tissues, allowing for accurate resection with negative surgical margins and favorable perioperative and postoperative outcomes. The advanced visualization capabilities of robotic systems, including 3D imaging and magnification, facilitate meticulous dissection and the preservation of vital structures [63].

#### 5.1.2. Blood Loss

A notable advantage of robot-assisted urachal surgery is its low intraoperative blood loss. Several studies have highlighted this benefit across various procedures. For example, Raynor et al. reported an average blood loss of <200 mL during robot-assisted surgeries for urachal anomalies [31], while Rivera et al. reported values of <225 mL [32]. Kim et al. observed comparable losses, ranging from 130 to 260 mL [28]. This clinically insignificant blood loss is primarily attributed to the enhanced precision and control provided by robotic systems, which improve hemostasis.

#### 5.1.3. Hospitalization Periods

Robot-assisted surgery is associated with shorter hospitalization periods. Studies have consistently shown that patients undergoing robot-assisted procedures experience minimal hospital and postoperative stays. Fode et al. reported a mean hospital stay of 1–2 days for patients undergoing robotic resection of urachal remnants [34]. Similarly, Spiess et al. documented a 4-day hospitalization following robot-assisted partial cystectomy for urachal adenocarcinoma [40]. These shorter hospital stays are attributed to the minimally invasive nature of robotic surgery, which reduces tissue trauma, decreases postoperative pain, and accelerates recovery.

#### 5.1.4. Complication Rates

The overall complication rates for robot-assisted urachal surgeries observed across the studies were minimal. Ahmed et al., in their case series, reported very few significant complications, with the primary issue being a bladder leak that required surgical repair [35]. Some studies suggest that robot-assisted surgery may be associated with lower complication rates compared to traditional open surgery, although outcomes can vary depending on the procedure type and patient-specific factors. Yong et al. reported no perioperative complications, including urinary leaks or bowel injuries, in their series of robot-assisted partial cystectomies [20]. Similarly, Rahmani et al. documented the successful treatment of an infected urachal cyst in a teenage patient with no significant postoperative complications, highlighting the safety and efficacy of the robotic approach in pediatric patients [16].

#### 5.1.5. Clinical and Oncological Outcomes

Patients who undergo RAUEPC typically experience positive clinical outcomes. These procedures result in shorter recovery times, reduced postoperative discomfort, and fewer surgical site infections, all of which contribute to higher patient satisfaction and lower healthcare costs due to the quicker resumption of daily activities [35,40]. Evaluating long-term oncological outcomes is essential to determine the efficacy of robot-assisted surgery for urachal tumors. Recent studies have confirmed the viability of the robotic approach in both benign and malignant cases. Advanced imaging techniques, such as intraoperative real-time imaging and augmented reality, have significantly improved the ability to achieve clear surgical margins. This is crucial in oncological surgeries, where complete removal of malignant tissue is necessary to minimize recurrence rates. Jiang et al. demonstrated that these techniques effectively achieved negative margins, reducing the likelihood of tumor recurrence [10]. Recurrence rates remain low, and overall survival rates are comparable to or even better than those of traditional surgical methods, particularly in younger patients and those with early-stage tumors [41].

#### 5.1.6. Efficacy and Safety

Although the short-term results are promising, long-term investigations are crucial to fully assess the efficacy and safety of robot-assisted surgeries for urachal tumors. Ongoing patient surveillance is necessary to evaluate the durability of surgical outcomes, recurrence rates, and potential delayed complications. Spiess et al. emphasized the importance of extended follow-up periods to collect critical data on the long-term success of these procedures [40]. Comprehensive long-term follow-up is essential to understand the true impact of robot-assisted surgery on patient survival and quality of life. Notably, the recurrence rate of adenocarcinoma remains low after robot-assisted surgery, according to various studies.

#### 5.1.7. Recovery and Quality of Life

Patients undergoing robot-assisted surgeries for urachal tumors generally experience quicker recovery and improved quality of life. Key advantages include reduced postoperative pain and a faster return to daily activities. Thiesfeldt et al. reported notable improvements in lower urinary tract symptoms in a young male patient following robotic excision of a urachal cyst, highlighting the benefits of this minimally invasive approach [61]. Additionally, the ability of robotic surgery to minimize visible scarring and maintain cosmetic outcomes is an important consideration for many patients.

### 5.2. Comparative Data with Conventional Surgical Techniques

#### 5.2.1. Feasibility

Open surgery is generally associated with a shorter operative time, averaging 87.8 min (range: 45–198 min), whereas laparoscopic approaches require a mean operative time of 145.9 min (range: 40–242 min), primarily due to the technical complexity of intracorporeal suturing and dissection [64]. In comparison with findings from the literature, RAUEPC demonstrates a mean operative time of 177.8 min (95% CI: 147.1–208.4 min), making it longer than both open and laparoscopic approaches. For comparison, laparoscopic radical cystectomy with extracorporeal urinary diversion has been reported with operative times ranging from 431 min (IQR: 355–499) [65].

Open surgery is associated with greater intraoperative blood loss, ranging from 50 to 110 mL, while laparoscopic procedures result in significantly lower blood loss, averaging 28.0 ± 6.4 mL [66]. In this context, RAUEPC reports an estimated blood loss of 85.4 mL (95% CI: 52.6–118.1 mL), which is higher than that observed in laparoscopic procedures but lower than in open surgery. Similarly, laparoscopic radical cystectomy has reported intraoperative blood loss ranging from 250 to 415 mL, which is significantly higher than the blood loss in RAUEPC [65].

Laparoscopic procedures carry a conversion rate of 5–10%, primarily due to intraoperative complications or challenges in achieving negative surgical margins [67]. In contrast, RAUEPC has not been associated with conversions to open surgery, suggesting comparable or superior feasibility in this regard.

#### 5.2.2. Efficacy

Both conventional approaches demonstrate comparable rates of complete excision, with negative margin rates ranging from 85% to 95% for laparoscopic surgery and 80% to 92% for open surgery [68]. In the analyzed cases, RAUEPC achieved a 100% rate of complete excision, indicating comparable or potentially superior efficacy.

The incidence of positive surgical margins is slightly higher in open procedures (8–10%) compared to laparoscopic surgery (5–7%), likely due to the enhanced precision afforded by laparoscopic magnified visualization [69]. Notably, RAUEPC reported no cases of R1 resections, suggesting a potentially lower risk of positive margins and improved oncologic outcomes.

#### 5.2.3. Safety

Open surgery is associated with a higher rate of intraoperative complications (10–15%) compared to laparoscopic surgery (5–8%) [70]. In this regard, RAUEPC demonstrated a complication rate of 10.04%, placing it between laparoscopic and open approaches in terms of intraoperative safety.

Laparoscopic surgery is linked to lower rates of wound infections and dehiscence (5–10%) compared to open surgery (15–20%) [70]. RAUEPC reported minor complications in 2.76% of cases and major complications in 8.28%, suggesting a lower complication rate than open surgery and comparable outcomes to laparoscopy.

Patients undergoing open surgery experience a longer postoperative hospital stay (5.2 ± 0.4 days) compared to those undergoing laparoscopic surgery (3.0 ± 0.5 days) [67]. RAUEPC demonstrated a mean hospital stay of 3.64 days, positioning it slightly above laparoscopic approaches but was significantly shorter than open surgery. For reference, mean hospital stay following laparoscopic radical cystectomy has been reported as 5 days (IQR: 1.25–9.75) [65].

Laparoscopic techniques are associated with a readmission rate of 3–5%, while open procedures exhibit higher readmission rates (7–10%), predominantly due to wound-related complications [15]. Notably, RAUEPC reported a readmission rate of only 0.69%, which is significantly lower than both conventional techniques, indicating a favorable safety profile.

#### 5.2.4. Short-Term Clinical Outcomes

Open surgery typically involves umbilical excision in 100% of cases, whereas laparoscopic surgery preserves the umbilicus in approximately 30–40% of cases [68]. RAUEPC demonstrated an umbilicus removal rate of 85.7%, making it more comparable to open surgery in this regard.

Lymphadenectomy is more commonly performed in open surgery (60–70%) than in laparoscopic procedures (40–50%), although lymph node positivity rates remain similar (15–20%) across both approaches [15]. RAUEPC demonstrated a lymphadenectomy rate of 33.3%, which is lower than that reported for open surgery but higher than for laparoscopy.

Both techniques achieve high rates of clear surgical margins and comparable histopathological outcomes, with adenocarcinoma detected in 40–50% of cases [66]. RAUEPC reported a malignancy rate of 33.79%, with 66.21% of cases being benign, reflecting a similar distribution of malignant and benign cases to those observed in conventional approaches.

#### 5.2.5. Long-Term Follow-Up Outcomes

The recurrence rate is slightly lower for laparoscopic surgery (10–15%) compared to open surgery (15–20%) [64]. RAUEPC demonstrated a recurrence rate of 2.07%, which is significantly lower than both conventional approaches. In studies analyzing radical cystectomy with orthotopic neobladder, recurrence rates ranged from 10% to 15%, showing a higher risk compared to RAUEPC [71].

Adjuvant therapy is required in approximately 20–30% of patients undergoing open surgery, compared to 15–25% of those treated with laparoscopic techniques [68]. In contrast, only 4.14% of RAUEPC cases required adjuvant therapy, indicating a substantially lower need for additional treatment compared to conventional approaches.

Both techniques demonstrate comparable long-term survival rates, ranging from 50% to 60% at five years, depending on tumor staging and margin status [69]. RAUEPC reported a median follow-up of 10.27 months, suggesting favorable oncologic control; however, longer-term data are required for comprehensive survival analysis. For comparison, patients undergoing orthotopic neobladder reconstruction following radical cystectomy had a reported five-year survival rate of 55% [71].

#### 5.2.6. Summary

In conclusion, while both open and laparoscopic approaches remain viable options for urachal excision and partial cystectomy, RAUEPC demonstrates potential advantages, including lower recurrence rates, shorter hospital stays than open surgery, and reduced complication rates. However, it may require a longer operative time compared to open surgery and slightly longer than laparoscopic surgery. Its possibly lower need for adjuvant therapy and reduced readmission rates suggest that it could be a favorable approach for selected cases.

### 5.3. Relation of Findings to Ongoing Discussions in Minimally Invasive Urologic Oncology and Global Surgical Practices

#### 5.3.1. Advantages of Robotic-Assisted Surgery in Urologic Oncology

The benefits of robotic-assisted surgery in urologic oncology have been extensively validated through systematic reviews and meta-analyses, demonstrating superior precision, reduced intraoperative blood loss, and lower complication rates compared to open and laparoscopic approaches. Wang et al. reported that robotic-assisted radical prostatectomy led to a 50% reduction in blood loss (median 500 mL vs. 1000 mL, *p* < 0.001), a lower transfusion rate (5.2% vs. 23.4%, *p* < 0.001), and a significantly shorter hospital stay (mean 3.1 vs. 6.5 days, *p* = 0.002) [72]. These findings are consistent with our results, where RAUEPC demonstrated a similar reduction in intraoperative blood loss and perioperative morbidity. Similarly, Cella et al. found that robotic-assisted radical cystectomy reduced transfusion rates by 57% (OR 0.43, 95% CI 0.30–0.61, *p* < 0.001) while increasing operative times by an average of 92 min (*p* < 0.001) [73]. Our findings align with this trend, as RAUEPC required a longer operative duration but provided benefits in reducing transfusion rates and overall morbidity. Si Ge et al. highlighted that robotic-assisted retroperitoneal lymph node dissection reduced intraoperative blood loss by 69% (WMD −0.69, 95% CI −1.07, −0.32, *p* < 0.05) and shortened hospital stays by 1.21 days (*p* < 0.05), findings comparable to those observed in our study, where patients undergoing RAUEPC experienced similar reductions in hospital stay and postoperative recovery times [74]. Patel et al. conducted a meta-analysis of randomized controlled trials (RCTs), demonstrating that robotic-assisted inguinal lymphadenectomy reduced overall complications by 45% (OR 0.41, 95% CI 0.24–0.72, *p* = 0.002), including significant reductions in wound infections (OR 0.15, *p* < 0.001) and skin necrosis (OR 0.12, *p* < 0.001) [75]. Our data similarly suggest that RAUEPC leads to lower rates of wound-related complications, reinforcing the broader advantages of minimally invasive robotic surgery in reducing perioperative morbidity.

#### 5.3.2. Economic and Global Implementation Considerations

The global adoption of robotic-assisted surgery is reshaping surgical practices, particularly in high-volume centers with access to advanced robotic platforms. Veccia et al. noted that robotic nephroureterectomy is increasingly accepted due to its reduced morbidity and comparable oncologic outcomes relative to open and laparoscopic techniques [76]. Our study supports these conclusions, as RAUEPC demonstrated a significant reduction in postoperative complications and improved surgical precision, making it a viable alternative in centers equipped with robotic platforms.

Baghli et al. emphasized the cost-effectiveness of robotic partial nephrectomy, demonstrating an 82% complication-free rate compared to 71% for open surgery (*p* < 0.001) while lowering the average cost per patient by EUR 1332 [77]. Similarly, Calpin et al. reported that robotic-assisted partial nephrectomy, despite higher initial costs, resulted in lower long-term complication rates, potentially offsetting the economic burden associated with robotic platforms [78].

While our study did not directly assess cost parameters, the reduction in complications and shorter hospitalization durations observed in RAUEPC cases suggest potential cost savings that warrant further investigation.

Despite these advantages, the adoption of robotic-assisted surgery in low- and middle-income countries (LMICs) remains challenging due to high costs and limited access. Mehta et al. outlined the potential benefits of robotic-assisted surgery in LMICs, including improved surgical precision, reduced physician workload, and lower infection rates [79]. However, key barriers such as financial constraints, the need for specialized training, and limited infrastructure hinder widespread implementation. In adrenal surgery, Gan et al. similarly reported that robotic-assisted adrenalectomy (RAA) had higher upfront costs than laparoscopic adrenalectomy but led to fewer postoperative complications, indicating that cost concerns should be weighed against long-term patient benefits [80]. Our findings highlight the need for cost-effectiveness analyses to better understand how RAUEPC can be integrated into resource-limited settings.

Lawrie et al. underscored the importance of strategic planning in robotic surgery adoption, emphasizing economic feasibility, institutional support, and workforce training as critical success factors [81]. Given the demonstrated perioperative benefits of RAUEPC in our study, policies facilitating wider access to robotic-assisted techniques should be explored, particularly in centers aiming to transition from open or laparoscopic approaches.

#### 5.3.3. Summary

This study corroborates broader advancements in minimally invasive urologic oncology, reinforcing the efficacy and safety of robotic-assisted surgery for urachal pathologies. The findings of RAUEPC align with the existing literature on robotic-assisted procedures, demonstrating advantages in surgical precision, reduced complications, and shorter hospital stays. However, disparities in global access to robotic technology necessitate policy initiatives, cost-reduction strategies, and expanded training programs to ensure equitable adoption. The integration of emerging technologies, including artificial intelligence-driven robotic platforms, holds promise for further enhancing surgical precision and improving oncologic outcomes. Future studies should focus on long-term oncologic data, cost-effectiveness analyses, and accessibility strategies to optimize the role of robotic-assisted approaches in global surgical practice.

### 5.4. Limitations of the Study

This study has several limitations that should be considered when interpreting the findings. A primary limitation is the reliance on case reports and small case series, which restricts the generalizability of the results and limits the potential for conducting a meta-analysis. This issue is compounded by the inclusion of non-standardized case definitions and varied reporting practices across studies. The study population introduces additional challenges due to its heterogeneity. The inclusion of both pediatric and adult populations results in variability in clinical presentation, surgical techniques, and outcomes. Reporting and methodological heterogeneity further exacerbate these issues. The studies included in this review report different metrics for the same outcomes, such as varying definitions of complications and follow-up durations, and the lack of uniform surgical, perioperative, and follow-up protocols limits comparability and introduces potential bias. Another critical limitation is the insufficient reporting of long-term outcomes. While short-term results are promising, long-term outcomes, including recurrence rates and overall survival, remain poorly documented. This gap prevents a comprehensive understanding of the procedure’s durability and oncological efficacy. Additionally, the small sample size in our single-center experience, involving only three cases, provides valuable insights but reflects a limited scope, introducing potential selection bias and affecting the robustness of the findings. The likelihood of publication bias is another concern, with studies reporting favorable outcomes more likely to be published. This skew in the evidence base limits the objectivity of conclusions. Economic considerations are notably absent from this study. The high costs associated with robotic surgery, including the acquisition and maintenance of robotic systems and the cost-effectiveness of procedures, have not been evaluated. These factors are critical for assessing the broader applicability of robot-assisted surgery. Additionally, patient-reported outcomes, such as satisfaction, quality of life, and cosmetic results, are underreported, limiting a holistic evaluation of the intervention’s impact. The statistical power of this study is constrained by the small number of cases, both in the literature and institutional data, reducing the ability to detect significant differences or trends and risking overinterpretation of findings. Surgeon expertise and the learning curve associated with robotic techniques also represent unaddressed variables. Variability in surgical proficiency could significantly impact outcomes, complicating comparisons across studies. Ethical and cultural variability among studies conducted in different regions adds another layer of complexity, as differing standards and expectations influence patient selection and treatment approaches. The absence of external validation through multicenter studies further limits the reliability and reproducibility of the findings.

### 5.5. Future Research Directions

RAUEPC offers numerous clinical benefits; however, challenges and opportunities remain for further advancement. A primary focus is the development of proficiency-based progression (PBP) training frameworks for surgeons undertaking these procedures, ensuring that they acquire and maintain the high level of skill necessary for optimal outcomes [82]. There is significant potential for creating structured, standardized curricula and dedicated mentorship programs specifically tailored to robotic urology [83,84,85,86]. The European Association of Urology emphasizes the need for standardized training to ensure that surgeons are equipped with the latest technological and procedural advancements [87]. Incorporating advanced simulation training, such as virtual reality (VR) environments and frequent skill assessments, into these programs would help maintain the highest standards of care, preparing surgeons to adapt effectively to ongoing technological innovations [88].

Technological limitations also present a significant avenue for research and development (R&D). While some advanced systems offer haptic force feedback, most robotic platforms lack this feature, limiting sensory experience. However, evidence suggests that haptic feedback significantly improves surgical outcomes by reducing force application, shortening completion time, and enhancing accuracy and success rates [89]. Collaborative efforts between clinicians and engineers are essential to enhance haptic feedback technologies across a broader range of systems, improving procedural precision and outcomes. Concurrently, efforts to reduce the costs associated with robotic systems would increase accessibility, particularly in resource-limited settings [90,91]. The continued development of simulation technology is equally important, as it provides a practical and safe platform for surgeons to refine their skills and adapt to new systems, addressing both training and technological gaps in a controlled environment [92].

To address these limitations, future research should prioritize studies that incorporate direct comparisons with laparoscopic and open surgical techniques. Stratifying populations by age (pediatric vs. adult) and pathology (benign vs. malignant) would improve the clarity of findings. Standardized reporting frameworks, such as the CONSORT and STROBE guidelines, should be utilized for consistent data collection and analysis.

Patient selection criteria also require further investigation to identify which patients would benefit most from robot-assisted versus traditional surgical approaches [93,94]. Clinical factors, such as tumor size, location, and patient health status, play a crucial role in determining the suitability of robotic surgery. Developing a more nuanced understanding of these criteria through targeted research could provide clearer guidelines, ultimately assisting clinicians in making more precise evidence-based recommendations and potentially reducing the risk of complications.

The assessment of long-term outcomes remains a critical area for future research. While short-term results are promising, extended follow-up studies are necessary to confirm the durability of the outcomes achieved through robotic surgery and to facilitate direct comparisons with traditional methods. To address these gaps, future research should prioritize prospective multicenter studies with larger sample sizes to provide robust data. Additionally, the inclusion of long-term follow-up data is essential for evaluating the durability of oncological and functional outcomes, recurrence rates, and survival. Cost-effectiveness analyses should be conducted to determine the economic viability of robotic procedures, while patient-reported outcomes should be included to assess satisfaction and quality of life.

Preliminary findings suggest that well-trained surgeons can achieve comparable or superior results with robotic techniques, underscoring the need for rigorous, long-term evaluations to substantiate these observations [95].

Finally, economic viability is an important consideration for the broader adoption of robotic surgery. While early studies suggest that robotic surgery may be more cost-effective than traditional methods, comprehensive analyses across diverse healthcare settings are required to confirm these findings [96]. Implementing cost-effective simulation training to improve surgical skills could reduce the costs associated with surgical errors, further supporting the economic feasibility of robotic procedures [97].

Multicenter, prospective trials with larger sample sizes and diverse populations are necessary to validate these findings. By addressing these issues, future research can provide more robust evidence to comprehensively assess the efficacy, safety, and cost-effectiveness of robot-assisted urachal procedures.

Exploring the potential of artificial intelligence and machine learning could significantly enhance surgical precision and patient outcomes [98,99,100]. AI-driven advancements, particularly in preoperative planning and real-time intraoperative decision-making, could further optimize procedural efficiency, support surgeons in achieving superior clinical outcomes, and improve overall quality of care.

## 6. Conclusions

This systematic review, combined with institutional experience, suggests that RAUEPC may be a feasible and safe approach for managing both benign and malignant urachal pathologies. The available data indicate promising short-term outcomes, including low intraoperative blood loss, a short mean hospital stay, acceptable complication rates, and no reported conversions to open surgery. The procedure appears to be effective in achieving complete resection with negative surgical margins, which is critical for reducing the risk of recurrence, particularly in malignant cases.

Short-term clinical outcomes, such as the consistent achievement of negative surgical margins, low complication rates, and favorable histopathological findings, suggest the potential efficacy of RAUEPC. Additionally, umbilical removal and lymphadenectomy were not universally performed, reflecting variability in clinical indications and patient presentations. However, these aspects require further evaluation in larger studies.

Long-term follow-up data remain limited, but the recurrence rates observed in both the literature and institutional cases seem low, supporting the potential oncological efficacy of this approach. The absence of metastatic cases in our series and the limited need for adjuvant therapy suggest the effectiveness of RAUEPC in appropriately selected cases. However, longer follow-up periods are essential to validate these observations.

Factors influencing outcomes, such as patient selection criteria, diagnostic accuracy, and surgical success predictors, were identified as key areas for optimization. Standardized protocols and evidence-based guidelines are necessary to enhance the consistency and reliability of outcomes.

These findings emphasize that while RAUEPC appears promising, larger prospective studies are needed to validate these results and fully assess its long-term safety, efficacy, and cost-effectiveness in managing urachal pathologies.

## Figures and Tables

**Table 1:** Sheldon staging system for urachal adenocarcinoma.

Stage I: Tumor confined to the urachus.
Stage II: Tumor invading the bladder.
Stage III: Local extension beyond the bladder.
IIIA: Tumor invasion into the abdominal wall. IIIB: Tumor invasion into the peritoneum. IIIC: Tumor invasion into other local structures.
Stage IV: Metastatic disease.
IVA: Regional lymph node metastasis. IVB: Distant metastasis.

**Table 2:** Currently recruiting and/or active clinical trials focusing on the oncological treatment of urachal adenocarcinoma (data from Clinicaltrials.gov).

NCT Number	Study Title	Study Status	Intervention
NCT00082706	Fluorouracil, Leucovorin, Gemcitabine, and Cisplatin in Treating Patients With Metastatic or Unresectable Adenocarcinoma	Active, Not Recruiting	5-FU, Leucovorin, Cisplatin, Gemcitabine
NCT05756569	Enfortumab Vedotin Plus Pembrolizumab for the Treatment of Locally Advanced or Metastatic Bladder Cancer of Variant Histology	Recruiting	Enfortumab Vedotin, Pembrolizumab
NCT04923178	A Multicenter Natural History of Urothelial Cancer and Rare Genitourinary Tract Malignancies	Recruiting	None (Observational)
NCT03866382	Testing the Effectiveness of Two Immunotherapy Drugs (Nivolumab and Ipilimumab) With One Anti-cancer Targeted Drug (Cabozantinib) for Rare Genitourinary Tumors	Recruiting	Cabozantinib, Ipilimumab, Nivolumab
NCT06638931	Agnostic Therapy in Rare Solid Tumors	Recruiting	Nivolumab

**Table 4:** Robot-assisted urachal excision and partial cystectomy: data from studies included in systematic review and institutional experience summary related to outcomes and follow-up.

Author (Year)	Institution (Country)	Volume (Number of Cases)	Reason for Surgery (Number of Cases)	Symptoms	Imaging Method	Cystoscopy Result	Preoperative TURBT	Lymph Node Involvement	Metastases	Umbilicus Removal	Lymphadenectomy	Complications	Hospital Stay (Days)	Histopathological Findings (Number of Results)	Robotic System	Operation Time (min.)	Console Time (min.)	Blood Loss (mL)	Patient Sex (M—Male, F—Female)	Patient Age	Follow-Up Duration (Months)	Follow-Up Cystoscopy	Follow-Up Cystoscopy Result	Follow-Up CT	Follow-Up CT Result	Adjuvant Therapy	Adjuvant Therapy Details
Madeb R. (2006) [25]	Rochester General Hospital (USA)	5	Urachal anomalies (remnants) (2), urachal adenocarcinoma (3)	Hematuria, irritative LUTS, dysuria	US, CT, MRI	Urachal submucosal mass, bladder dome tumor	No	No	No	Yes	Yes	Small bowel perforation, postoperative repair	2 (3 patients), 14 (1 patient), 6 (1 patient)	Benign diverticulum (1), adenocarcinoma (3), leiomyoma (1)	da Vinci	120–480	Not Available (N/A)	25–300	3 M 2 F	22–68	8	Yes	Normal	Yes	No recurrence	No	N/A
Yamzon J. (2008) [39]	University of Southern California (USA)	1	Urachal cyst	Midline abdominal pain	CT	N/A	No	No	No	Yes	No	None	N/A	Benign urachal cyst with acute and chronic inflammation	da Vinci	N/A	N/A	N/A	F	4	N/A	N/A	N/A	N/A	N/A	No	N/A
Spiess P.E. (2009) [40]	H. Lee Moffitt Cancer Center (USA)	1	Urachal adenocarcinoma	Hematuria, mucosuria	CT	Bladder dome tumor	Yes	No	No	Yes	Yes	None	4	pT2N0Mx adenocarcinoma with negative margins	N/A	300	N/A	150	M	55	N/A	N/A	N/A	N/A	N/A	No	N/A
Nayyar R. (2009) [26]	All India Institute of Medical Sciences (India)	3	Urachal adenocarcinoma	Hematuria	US, CT	Bladder dome tumor, margins marked	Yes	No	No	Yes	Yes	None	3	Urachal adenocarcinoma, margins free (3)	da Vinci S	182	N/A	<100	Mixed (non-specified)	N/A	8	Yes	Normal	N/A	N/A	No	N/A
Correa J.J. (2009) [27]	H. Lee Moffitt Cancer Center (USA)	2	Urachal adenocarcinoma	Hematuria, mucosuria, pain	CT	Bladder dome tumor	Yes	Yes (1 patient)	No	Yes	Yes	None	4 (1 patient), 7 (1 patient)	pT2NxMx adenocarcinoma, pT3N1Mx adenocarcinoma	N/A	300–354	N/A	100–150	M	53–55	<3	N/A	N/A	N/A	N/A	Yes	Cisplatin, 5-fluorouracil (1 patient)
Allaparthi S. (2010) [41]	University of Massachusetts Medical School (USA)	1	Urachal adenocarcinoma	Hematuria	CT, MRI	Bladder dome tumor	Yes	No	No	Yes	No	Bowel obstruction requiring resection (readmission)	2	Invasive adenocarcinoma, margins free	da Vinci	165	N/A	20	M	24	6	Yes	Normal	Yes	No recurrence	No	N/A
Kim D.K. (2010) [28]	Hanyang University (Korea)	4	Urachal cyst (2), patent urachus (1), urachal cystadenoma (1)	Hematuria, dysuria, mucosuria	CT	Bladder dome mass	No	No	No	Yes	No	None	4–7	Urachal cyst (2), patent urachus (1), urachal cystadenoma (1)	da Vinci S	130–260	70–150	20–250	2 M 2 F	45–65	1	N/A	N/A	N/A	N/A	No	N/A
Lee H.E. (2010) [29]	Seoul National University Hospital (Korea)	2	Urachal cyst	Gross hematuria, lower abdominal pain	CT	Urachal cyst with inflammation	No	No	No	Yes	No	None	1	Urachal cyst with non-specific inflammation	N/A	220–225	N/A	Minimal	1 M 1 F	43–47	N/A	No	N/A	No	N/A	No	N/A
Tadtayev S. (2011) [30]	Lister Hospital (UK)	4	Urachal adenocarcinoma	N/A	N/A	Bladder dome tumor	Yes	Yes (3 patients)	Yes (1 patient)	Yes	Yes	None	4	pT2-pT3 adenocarcinoma, villous adenoma	N/A	150–240	70–170	20–250	N/A	N/A	N/A	Yes	Normal (all 3 cases with curative intent)	Yes	No recurrence (all 3 cases with curative intent)	No	N/A
Raynor M. (2011) [31]	University of North Carolina (USA)	12	Urachal adenocarcinoma (4), symptomatic urachal cyst/sinus (8)	N/A	N/A	N/A	N/A	N/A	N/A	Yes	N/A	None	1–3	N/A	N/A	79–200	N/A	25–75	7 M 5 F	42.1 (mean)	N/A	N/A	N/A	N/A	N/A	No	N/A
Kosanovic R. (2014) [42]	Baptist Health South Florida (USA)	1	Urachal adenocarcinoma	Recurrent UTIs	US, MRI	Normal	No	No	No	Yes	No	None	3	Well-differentiated mucinous adenocarcinoma, muscularis propria involvement	da Vinci	N/A	N/A	N/A	F	53	N/A	Yes	Normal	N/A	N/A	No	N/A
Rivera M. (2015) [32]	Mayo Clinic (USA)	11	urachal cyst (5), urachal remnant (3), fibrovascular necrotizing granuloma (1), urachal cyst with colonic metaplasia (1), urachal cyst with fibrosis (1)	Umbilical drainage, abdominal pain, infection	CT, MRI	N/A	No	No	No	No	No	UTI requiring antibiotics	1–2	urachal cyst (5), urachal remnant (3), fibrovascular necrotizing granulomatous tissue (1), urachal cyst with colonic metaplasia (1), urachal cyst with fibrosis (1)	N/A	51–224	N/A	5–400	7 M 4 F	12–72	1–18 15.5 (mean	N/A	N/A	N/A	N/A	No	N/A
Aoun F. (2015) [43]	Jules Bordet Institute (Belgium)	1	Urachal adenocarcinoma	Gross hematuria	US, MRI	Bladder dome mass (small)	Yes	No	No	Yes	Yes	None	4	Moderately differentiated mucinous colloid adenocarcinoma, pT2b stage	da Vinci SI	N/A	N/A	N/A	F	47	3	Yes	Normal	Yes	No recurrence	No	N/A
James K. (2015) [33]	Lister Hospital (UK)	8	Urachal adenocarcinoma	Hematuria, dysuria	CT, MRI	Bladder dome mass	Yes	Yes	Yes (1 patient)	Yes	Yes	None	4	Primary urachal adenocarcinoma of bladder, no positive margins	da Vinci S	130–240	70–170	50	5 M 3 F	49–63	32	Yes	Normal (8/8 patients)	Yes	No recurrence (7/8 patients)	Yes	Oxaliplatin and Capecitabine (1 patient)
Williams C.R. (2015) [44]	University of Florida (USA)	1	Urachal adenocarcinoma	Abdominal pain, hematuria	CT, PET scan	N/A	Yes	No	No	Yes	Yes	None	1	Urachal mucinous adenocarcinoma, negative surgical margin, no lymph node involvement	da Vinci	300	N/A	5	F	20	N/A	N/A	N/A	N/A	N/A	No	N/A
Fode M. (2016) [34]	Zealand University Hospital (Denmark)	9	Urachal Remnants	Hematuria, umbilical secretion, UTI	CT	Urachal remnant mass (1 case), urachal remnant ducts (5 cases)	No	No	No	Yes	No	Fascia rupture (3), bleeding spleen (1)	1–2	well-differentiated adenocarcinoma (1), benign lesions (8)	N/A	90–120	N/A	N/A	5 M 4 F	15–73	36	Yes	Normal	Yes	No recurrence	No	N/A
Dababneh H. (2016) [18]	Sant’Orsola Malpighi, Bologna (Italy)	1	Urachal acinar adenocarcinoma	Hematuria	US, CT	Bladder dome tumor	Yes	No	No	Yes	Yes	None	3	pT3b acinar adenocarcinoma with negative surgical margins	N/A	300	250	<50	M	55	N/A	N/A	N/A	N/A	N/A	No	N/A
Kilday P.S. (2016) [7]	Kaiser Permanente Los Angeles (USA)	1	Urachal cyst	Dyspareunia, dysorgasmia, abdominal pain	CT, MRI	Normal	No	No	No	Yes	No	None	1	No malignancy	N/A	N/A	N/A	N/A	F	29	12	No	N/A	No	N/A	No	N/A
Shepler R. (2016) [45]	Eastern Virginia Medical School (USA)	1	Urachal hamartoma	Dysuria, urinary frequency, nocturia	CT	Bladder dome mass	Yes	No	No	No	No	None	1	Urachal hamartoma, benign	N/A	150	N/A	100	F	30	3	No	N/A	No	N/A	No	N/A
Ahmed H. (2017) [35]	Cohen Children’s Medical Center (USA)	16	Umbilical drainage (5), infections (7), umbilical drainage and infection (2), incidental findings (3)	Umbilical drainage, infection	US	Normal	Yes	No	No	Yes	No	Bladder leakage (1)	1–2	Chronic inflammation, no malignancy	N/A	107 (mean)	N/A	N/A	10 M 6 F	0.8–16.5	9–21	N/A	N/A l	N/A	N/A	No	N/A
Chen A. (2017) [46]	Albany Medical College (USA)	1	Urachal villous adenoma	Mucoid discharge, dysuria, hematuria	CT, cystogram	Urachal mass, mucous discharge	No	No	No	Yes	No	None	1	Villous adenoma with papillary fronds and fibrovascular cores, no malignancy	N/A	N/A	N/A	N/A	F	47	N/A	Yes	Normal	N/A	N/A	No	N/A
Fedelini P. (2018) [19]	A.Cardarelli Hospital, Dept. of Urology (Italy)	1	Mucinous urachal adenocarcinoma	Gross hematuria, dysuria	MRI	Bladder dome solid mass	Yes	Yes	No	Yes	Yes	None	4	Poorly differentiated adenocarcinoma, 2/17 lymph nodes positive for metastases	N/A	120	N/A	Minimal	M	40	6	Yes	Normal	Yes	No recurrence	Yes	Enrolled in strict follow-up protocol
Stillings S. (2018) [1]	Ohio State University Wexner Medical Center (USA)	1	Urachal remnant	Dysuria, perineal pain, hematuria	CT urogram	Bladder dome tumor (large)	Yes	No	No	Yes	No	None	3	Chronic inflammation, negative for malignancy	N/A	N/A	N/A	N/A	M	62	N/A	No	N/A	No	N/A	No	N/A
Proskura A. (2018) [47]	Sechenov University (Russia)	1	Mucinous urachal adenocarcinoma	Gross hematuria	MRI	Bladder dome mass (1 cm)	No	Yes	No	Yes	Yes	None	N/A	Mucinous adenocarcinoma invading submucosa, 1/23 lymph nodes positive	N/A	N/A	N/A	N/A	F	34	6	No	N/A	Yes	No recurrence	Yes	6 courses of 5-fluorouracil and cisplatin
Yong J. (2020) [36]	Singapore General Hospital (Singapore)	9	Urachal adenocarcinoma (3), benign urachal nodules (6)	Gross hematuria	CT	Solid urachal lesion	No	No	No	Yes (4 patients)	No	Urosepsis (1), acute urinary retention (1)	2	Urachal adenocarcinoma (3), benign nodules (6)	da Vinci Xi	190 (mean)	N/A	50	8 M 1 F	44–64	6	No	N/A	No	N/A	No	N/A
George R. (2021) [48]	Albany Medical Center (USA)	1	Urachal inflammatory myofibroblastic tumor	Abdominal pain, dysuria	US, CT	Mixed solid/cystic mass	Yes	No	No	Yes	No	None	2	Urachal inflammatory myofibroblastic tumor with ALK gene rearrangement	N/A	N/A	N/A	N/A	F	27	5	No	N/A	No	N/A	No	N/A
Connor J. (2021) [49]	Medical University of South Carolina (USA)	1	Mucinous cystadenocarcinoma of urachus	Frequency, urgency	CT, MRI	Bladder wall thickening	No	No	No	No	No	None	1	Mucinous cystadenocarcinoma, no invasion	N/A	N/A	N/A	N/A	M	67	12	No	N/A	Yes	No recurrence	No	N/A
Lough C.P. (2021) [50]	University of Missouri School of Medicine (USA)	1	Calcified urachal cyst	Gross hematuria	CT urogram	Normal	No	No	No	No	No	None	1	Calcified urachal cyst, no malignancy	N/A	N/A	N/A	N/A	F	50	N/A	N/A	N/A	N/A	N/A	N/A	N/A
Abou Zahr R. (2021) [51]	Jules Bordet Institute, Brussels (Belgium)	1	Mucinous urachal adenocarcinoma	Gross hematuria	CT, MRI	Mucinous lesion at bladder dome	Yes	No	No	Yes	No	None	5	Mucinous adenocarcinoma, pT3bNx	N/A	N/A	N/A	N/A	F	49	12	No	N/A	Yes	No recurrence	No	N/A
Arena S. (2021) [52]	University of Messina (Italy)	1	Urachal cyst	Suprapubic abdominal pain	MRI	Supra-vesical cyst	No	No	No	Yes	No	None	7	Benign urachal cyst, no malignancy	N/A	N/A	N/A	N/A	F	15	3	No	N/A	No	N/A	No	N/A
Osumah T.S. (2021) [37]	Mayo Clinic (USA)	14	Urachal remnant (9), cyst (3), patent urachus (2)	Abdominal pain, fever, UTI, umbilical drainage	US, CT	Normal	No	No	No	Yes (some cases)	No	UTI (1), persistent abdominal pain (1)	1	Benign findings, no malignancy	da Vinci Xi	133 (median)	N/A	Minimal	9 M 5 F	2–16	0.25	No	N/A	No	N/A	No	N/A
Shin H.B. (2021) [53]	Eulji University Hospital (Korea)	1	Eosinophilic cystitis with infected urachal cyst	Gross hematuria, fever, dysuria, suprapubic pain	CT	Raspberry-like mass at bladder dome	Yes	No	No	Yes	No	None	7	Eosinophilic cystitis with heavy eosinophilic infiltration and infected urachal cyst	N/A	N/A	N/A	N/A	M	59	23	Yes	Normal	Yes	No recurrence	No	N/A
Park J.J. (2021) [54]	Soonchunhyang University Hospital (Korea)	1	Large urachal adenocarcinoma	No symptoms	CT	Lobulated mass at bladder dome	Yes	No	No	Yes	No	None	14	Well-differentiated mucinous adenocarcinoma, negative margins	da Vinci Xi	150	N/A	50	M	71	3	No	N/A	Yes	No recurrence	No	N/A
Jiang J.Y. (2022) [10]	Nepean Hospital, Dept. of Nuclear Medicine (Australia)	1	Mucinous urachal adenocarcinoma	Dysuria, urgency, macrohematuria	PET/CT, MRI	Mixed solid/cystic mass with calcification	No	No	No	Yes	Yes	None	7	Mucinous adenocarcinoma, invasion through bladder wall	N/A	N/A	N/A	N/A	F	24	N/A	N/A	N/A	N/A	N/A	N/A	N/A
Perez D. (2022) [11]	Shaare Zedek Medical Center, Jerusalem (Israel)	9	Urachal cyst (5), sinus (2), diverticulum (1), patent urachus (1)	Umbilical discharge, abdominal pain, hematuria, recurrent UTI	US, CT, MRI, VCUG	Urachal mass	No	No	No	Yes (5 patients)	No	Grade IIIA (1) infected hematoma, Grade IIIB (1) abscess reoperation	1–7	6 cases with urothelium, including 2 with necrotizing granuloma; 3 cases without epithelium	N/A	52–140	N/A	N/A	6 M 3 F	0–37	N/A	N/A	N/A	N/A	N/A	No	N/A
Stokkel L.E. (2022) [38]	Netherlands Cancer Institute (Netherlands)	8	Urachal adenocarcinoma	Gross hematuria, abdominal pain	CT, MRI	Mass at bladder dome	No	Yes (3 patients)	No	Yes	Yes (3 patients)	Grade V (1) arterial occlusion, Grade III (1) urinary leakage	2–8	Adenocarcinoma, stages pT2–pT4, 3 cases with positive lymph nodes	N/A	90–180	N/A	N/A	5 M 3 F	42–79, 60 (mean)	25–56 31 (mean)	Yes	normal (1 patient), local recurrence (1 patient)	Yes	port-site recurrence (2 patients)	Yes	external radiotherapy and brachytherapy (1 patient), palliative chemotherapy—not specified (1 patient)
Kochvar A.P. (2023) [55]	Kansas City Urology Care, Kansas City (USA)	1	Low-grade urachal cystadenoma with calcification	Left lower quadrant pain	US, MRI	Extrinsic bladder compression	No	No	No	No	No	None	1	Low-grade mucinous cystadenoma with calcified mucin	N/A	N/A	N/A	N/A	F	57	N/A	Yes	Normal	Yes	No recurrence	No	N/A
Bogaerts Q. (2023) [56]	Ziekenhuis Oost-Limburg, Genk (Belgium)	1	Mucinous cystic tumor of low malignant potential	No symptoms	PET-CT	Mass at bladder dome	No	No	No	Yes	No	None	1	Mucinous cystic tumor with low malignant potential	da Vinci Xi	75	N/A	Minimal	F	59	6	No	N/A	Yes	No recurrence	No	N/A
Kunitsky K.S. (2024) [57]	Kansas City Urology Care, Kansas City (USA)	1	Symptomatic urachal remnant	Dysuria, urinary frequency, intermittent hematuria, right flank pain	CT	Bladder wall thickening	No	No	No	Yes	No	None	1	Urachal remnant, benign (no malignancy)	da Vinci SP	135	N/A	10	F	34	1	No	N/A	No	N/A	No	N/A
Hemal S. (2024) [58]	University of Southern California (USA)	1	Urachal adenocarcinoma	Hematuria, mucosuria	CT, MRI	Solitary tumor at bladder dome	Yes	Yes (bilateral)	No	Yes	Yes	None	1	Muscle-invasive adenocarcinoma, pT2b, negative margins	da Vinci SP	100	N/A	20	M	41	N/A	N/A	N/A	N/A	N/A	No	N/A
Hamasaki S. (2024) [59]	Saitama Medical University (Japan)	1	Urachal remnant	No symptoms	MRI	Protruding lesion with normal mucosa	No	No	No	Yes	No	None	6	Urachal remnant with normal urothelium	da Vinci Xi	N/A	N/A	Minimal	F	55	N/A	N/A	N/A	N/A	N/A	No	N/A
Elsheikh M. (2024) [60]	Royal Bournemouth Hospital, Bournemouth (UK)	1	Transmural bladder leiomyoma invading urachal remnant	Gross hematuria, dysuria	CT, MRI	4 cm mass at bladder dome	Yes	No	No	Yes	No	None	2	Infarcted bladder leiomyoma, no malignancy	N/A	N/A	N/A	N/A	M	29	N/A	No	N/A	No	N/A	No	N/A
Thiesfeldt D.L. (2024) [61]	University of Central Florida College of Medicine, Nemours Children’s Hospital (USA)	1	Large urachal cyst	Lower urinary tract symptoms, falsely elevated post-void residual	US, MRI	Normal	No	No	No	No	No	None	1	Urachal cyst with no malignancy	N/A	N/A	N/A	Minimal	M	11	N/A	No	N/A	No	N/A	No	N/A
Rich J.M. (2024) [62]	NYU Langone Health, NYU School of Medicine (USA)	1	Recurrent urachal cyst	Umbilical pain, umbilical drainage	CT, MRI	Cyst with rim-enhancing fluid collection	No	No	No	Yes	No	None	1	Urachal cyst, no malignancy	N/A	N/A	N/A	Minimal	M	24	N/A	N/A	N/A	N/A	N/A	No	N/A
Our work	Multidisciplinary Hospital in Warsaw-Miedzylesie (Poland)	3	Suspected urachal tumor (2), urachal tumor (1)	Suprapubic pain, hematuria	CT	Tumor at the bladder dome (2 cases), normal (1 case)	No	No	No	No	No	Gade II: red blood cell concentrates transfusion (1)	6.33 (mean) 2.66 postoperatively (mean)	Benign findings, no malignancy (2), mucinous cystadenocarcinoma (1)	da Vinci X	85.33 (mean)	57.66 (mean)	216.66 (mean)	3 M	52.66 (mean)	10.66 (mean)	Yes (1 patient)	Normal (cT0)	Yes (2 patients)	No recurrence	No	N/A

**Table 5:** Robot-assisted urachal excision and partial cystectomy: detailed data from single-center experience related to outcomes and follow-up.

Surgeon (Date)	Institution (Country)	Pre- and Postoperative Hemoglobin (mmol/L)	Reason for Surgery	Symptoms	Imaging Method	Cystoscopy Result	Preoperative TURBT	Lymph Node Involvement	Metastasis	Umbilicus Removal	Lymphadenectomy	Complications	Hospital Stay (Days)	Histopathological Findings	Robotic System	Operation Time (Min.)	Console Time (Min.)	Blood Loss (mL)	Patient Sex	Patient Age	Follow-Up Duration (Months)	Follow-Up Cystoscopy	Follow-Up Cystoscopy Result	Follow-Up CT	Follow-Up CT Result	Adjuvant Therapy	Adjuvant Therapy Details
Drobot RB 15 December 2022	Multidisciplinary Hospital in Warsaw-Miedzylesie (Poland)	9.9 ⟶ 8.4	Suspected urachal tumor on CT (contrast-enhanced tissue mass)	Chronic suprapubic pain	CT	Normal	No	No	No	No	No	II Clavien Dindo: red blood cell concentrate transfusion	7 (3 postoperatively)	Tissue fragment measuring 13 × 5 cm, consisting of adipose tissue, and an adjacent cohesive element measuring 3 × 2 × 5 cm. The obfuscated material is not very legible. Urachus without tumor.	da Vinci X	90	55	450	Male	44	20	No	Not Applicable (N/A)	Yes	No evidence of pathology	No	N/A
Drobot RB 19 October 2023	Multidisciplinary Hospital in Warsaw-Miedzylesie (Poland)	9.5 ⟶ 8.4	Urachal tumor Shelodon IIIA stage	Suprapubic pain, hematuria	CT	Tumor at the dome of the bladder	No	No	No	No	No	None	5 (2 postoperatively)	Cystadenocarcinoma mucinosum lesion excised completely (R0).	da Vinci X	85	50	150	Male	66	10	Yes	Normal (cT0)	Yes	No evidence of recurrence (N0, M0)	No	N/A
Drobot RB 12 September 2024	Multidisciplinary Hospital in Warsaw-Miedzylesie (Poland)	9.4 ⟶ 9.1	Suspected urachal tumor on CT	Microscopic hematuria	CT	Tumor at the dome of the bladder	No	No	No	No	No	None	7 (3 postoperatively)	The examined material includes samples of the patent urachus with focal, moderately abundant chronic inflammatory infiltrates; no neoplastic tissue is observed	da Vinci X	81	68	50	Male	48	2	No	N/A	No	N/A	No	N/A

**Table 6:** Detailed overview of identified symptoms in patients undergoing RAUEPC for benign and malignant urachal pathologies in the included studies.

Symptoms	Count (Number)	Percentage (%)
Hematuria	26	29.89
Abdominal pain	16	18.39
Dysuria	12	13.79
UTI	8	9.20
Irritative LUTS	8	9.20
Umbilical drainage/discharge	6	6.90
Mucosuria	5	5.75
No symptoms	3	3.45
Dyspareunia/dysorgasmia	2	2.30
Obstructive LUTS	1	1.15

**Table 7:** Detailed overview of identified cystoscopy descriptive results among patients undergoing RAUEPC for benign and malignant urachal pathologies in the included studies.

Cystoscopy Results	Count (Number)	Percentage (%)
Bladder dome mass/finding	20	40.38
Normal	6	11.54
Urachal remnant ducts	5	9.62
Bladder wall thickening	2	3.85
Urachal mass	2	3.85
Extrinsic bladder compression	1	1.92
Mixed solid/cystic mass	1	1.92
Mixed solid/cystic mass with calcification	1	1.92
Protruding lesion with normal mucosa	1	1.92
Solid urachal lesion	1	1.92
Supra-vesical cyst	1	1.92
Urachal cyst with inflammation	1	1.92
Cyst with rim-enhancing fluid collection	1	1.92
Urachal remnant mass	1	1.92
Urachal submucosal mass	1	1.92
Margins marked	1	1.92
Mucous discharge	1	1.92

**Table 8:** Identified complications (Clavien–Dindo) across the studies on RAUEPC for both benign and malignant urachal pathologies.

Category	Count (Number)	Percentage (%)	Details (Number)	Detailed Percentage (%)
No Complications	129	88.96	-	-
Grade I	1	0.69	Persistent abdominal pain (1)	0.69
Grade II	3	2.07	UTI requiring antibiotics (2) Acute urinary retention (1)	1.38 0.69
Grade IIIA	3	2.07	Bladder/urinary leakage (2) Infected hematoma (1)	1.38 0.69
Grade IIIB	7	4.83	Fascia rupture (3) Bowel obstruction requiring resection (1) Abscess reoperation (1) Small bowel perforation requiring surgical repair (1) Bleeding spleen (1)	2.07 0.69 0.69 0.69 0.69
Grade IVa	1	0.69	Urosepsis (1)	0.69
Grade V	1	0.69	Arterial occlusion (death) (1)	0.69

**Table 9:** Evidence-based guidance for diagnostic and therapeutic decisions in RAUEPC for benign and malignant urachal pathologies.

Key Finding	Statistical Significance	Level of Evidence GRADE Approach	Recommendation Strength	Recommendation
Imaging method choice is significantly influenced by symptoms. Gross hematuria leads to CT/MRI use, while non-specific symptoms often result in ultrasound.	χ^2^ = 1761 *p* < 2.2 × 10^−16^	Low Retrospective cohort studies; low risk of bias but limited by imprecision and study design limitations.	Strong	Advanced imaging modalities (CT/MRI) should be prioritized for patients with gross hematuria.
Imaging method varies depending on adjuvant therapy. CT was more common in patients receiving therapy.	χ^2^ = 38.51 *p* = 2.42 × 10^−6^	Low Small retrospective studies and case reports; indirect evidence with inconsistent reporting.	Weak	CT should be performed before initiating adjuvant therapy for accurate staging.
Imaging preferences differ by age. Younger patients (<30 years) favor ultrasound, older patients (>50 years) favor CT/MRI.	χ^2^ = 1088.7 *p* < 2.2 × 10^−16^	Low Retrospective data; consistent findings but lacking prospective validation.	Weak	Imaging modalities should consider the patient’s age, with CT/MRI recommended for older patients due to the higher likelihood of malignant pathologies.
Symptom presentation differs by sex: males are more likely to present with gross hematuria, females with non-specific symptoms	χ^2^ = 74.9 *p* < 0.05	Low Case series and small observational studies; evidence consistent but imprecise	Weak	Consider advanced imaging for males with gross hematuria to exclude malignancy.
Males undergo CT more frequently, while females favor ultrasound evaluations.	χ^2^ = 33.6 *p* < 0.05	Low Retrospective studies; limited subgroup sizes and lack of prospective validation.	Weak	The choice of imaging modality should be guided by clinical indications and symptoms, without being influenced solely by the patient’s sex.
Cystoscopy findings are more suggestive of malignancy in males compared to females.	χ^2^ = 65.6 *p* < 0.05	Low Observational studies and case series; statistically significant but constrained by study quality.	Strong	Male patients with suspicious or undetermined findings on cystoscopy should be prioritized for advanced diagnostic work-up to exclude malignancy.
Adjuvant therapy administration is influenced by sex, with males more likely to receive therapy.	χ^2^ = 7.2 *p* < 0.05	Low Retrospective observational studies; indirect evidence with small sample sizes.	Weak	Evaluate patient characteristics thoroughly when considering adjuvant therapy.
Symptom presentation varies significantly across age groups. Younger patients (<30 years) present non-specific symptoms, while older patients (>50 years) exhibit gross hematuria.	χ^2^ = 6727 *p* < 0.05	Low Retrospective study design with limited precision and absence of prospective comparative studies.	Weak	Consider age-related symptom variability when planning diagnostic evaluations.
Younger patients undergo surgery for benign conditions; older patients for malignancies.	χ^2^ = 6700 *p* < 0.05	Low Case series and retrospective cohort studies; limited by study design and data precision.	Weak	Surgical decisions should consider age-related pathology trends, ensuring malignancies in older patients are appropriately prioritized when clinically indicated.
Positive cystoscopy findings are strongly associated with adjuvant therapy administration, and the reason for adjuvant therapy is strongly linked to cystoscopy results.	χ^2^ = 208.7 *p* < 2.2× 10^−16^ χ^2^ = 1117 *p* < 2.2 × 10^−16^	Low Retrospective data; statistically robust but constrained by limited data.	Strong	Cystoscopy outcomes should guide decisions regarding adjuvant therapy, with positive findings prompting further diagnostic and therapeutic considerations.
Adjuvant therapy administration differs by age, with middle-aged patients (50–65 years) most likely to receive it.	χ^2^ = 793.62 *p* < 0.05	Low Retrospective observational studies; indirect evidence with imprecision due to small sample sizes.	Weak	Adjuvant therapy decisions in malignant cases should be based on staging and pathology, while acknowledging that middle-aged patients (50–65 years) are more likely to require it due to disease characteristics.

## Data Availability

All data underlying the findings of this study are available within this publication. Summary data derived from hospital patients at the Multidisciplinary Hospital in Warsaw-Miedzylesie are included in anonymized form to ensure confidentiality. Due to ethical and legal restrictions related to data protection regulations, raw patient data cannot be shared publicly. Requests for further information may be directed to the corresponding author.

## References

[1] Stillings S., Merrill M. (2018). Post TURBT Discovery of Urachal Remnant in a 62-Year-Old Man. Urology.

[2] Mccollum M.O., Macneily A.E., Blair G.K. (2003). Surgical implications of urachal remnants: Presentation and management. J. Pediatr. Surg..

[3] Stopak J.K., Azarow K.S., Abdessalam S.F., Raynor S.C., Perry D.A., Cusick R.A. (2015). Trends in surgical management of urachal anomalies. J. Pediatr. Surg..

[4] Iuchtman M., Rahav S., Zer M., Mogilner J., Siplovich L. (1993). Management of urachal anomalies in children and adults. Urology.

[5] Siefker-Radtke A. (2012). Urachal adenocarcinoma: A clinician’s guide for treatment. Semin. Oncol..

[6] Schubert G.E., Pavkovic M.B., Bethke-Bedürftig B.A. (1982). Tubular urachal remnants in adult bladders. J. Urol..

[7] Kilday P.S., Finley D.S. (2016). Robot-Assisted Excision of a Urachal Cyst Causing Dyspareunia and Dysorgasmia: Report of a Case. J. Endourol. Case Rep..

[8] Naiem M.E.A., Mohammed N.I.M., Mohammed R. (2022). Infected urachal sinus with de novo stone and peritonism in a young athlete adult: A case report. Int. J. Surg. Case Rep..

[9] Hassan S., Koshy J., Sidlow R., Leader H., Horowitz M. (2017). To excise or not to excise infected urachal cysts: A case report and review of the literature. J. Pediatr. Surg. Case Rep..

[10] Jiang J.Y., Kang C., Jackson S., Jeffery N., Winter M., Le K., Mansberg R. (2022). A rare case of urachal mucinous adenocarcinoma detected by 18F-FDG PET/CT and MRI. Radiol. Case Rep..

[11] Perez D., Neeman B., Kocherov S., Jaber G., Armon Y., Zilber S., Chertin B. (2022). Current management of the urachal anomalies (UA). Lessons learned from the clinical practice. Pediatr. Surg. Int..

[12] Das J.P., Vargas H.A., Lee A., Hutchinson B., O’Connor E., Kok H.K., Torreggiani W., Murphy J., Roche C., Bruzzi J. (2020). The urachus revisited: Multimodal imaging of benign & malignant urachal pathology. Br. J. Radiol..

[13] Orsini A., Bignante G., Lasorsa F., Bologna E., Mossack S.M., Pacini M., Marchioni M., Porpiglia F., Lucarelli G., Schips L. (2024). Urachal Carcinoma: Insights From a National Database. Clin. Genitourin. Cancer.

[14] Sheldon C.A., Clayman R.V., Gonzalez R., Williams R.D., Fraley E.E. (1984). Malignant urachal lesions. J. Urol..

[15] Loizzo D., Pandolfo S.D., Crocerossa F., Guruli G., Ferro M., Paul A.K., Imbimbo C., Lucarelli G., Ditonno P., Autorino R. (2022). Current Management of Urachal Carcinoma: An Evidence-based Guide for Clinical Practice. Eur. Urol. Open Sci..

[16] Rahmani P., Ashjaee B., Zamani F., Sharifi P. (2022). Infected urchus cyst in a teenage girl. Urol. Case Rep..

[17] Pandolfo S.D., Cilio S., Aveta A., Wu Z., Cerrato C., Napolitano L., Lasorsa F., Lucarelli G., Verze P., Siracusano S. (2024). Upper Tract Urothelial Cancer: Guideline of Guidelines. Cancers.

[18] Dababneh H., Gandaglia G., De Groote R., Geurts N., Schatteman P., D’Hondt F., De Naeyer G., Zazzara M., Novara G., Schiavina R. (2016). V32 Robot-assisted partial cystectomy for the treatment of urachus acinar adenocarcinoma. Eur. Urol. Suppl..

[19] Fedelini P., Chiancone F., Fedelini M., Fabiano M., Meccariello C. (2018). Robot-assisted laparoscopic partial cystectomy, urachal resection and pelvic lymphadenectomy for urachal adenocarcinoma. Eur. Urol. Suppl..

[20] Yong J., Law Z.W., Chen K., Sim S.P.A., Lee L.S., Yuen S.P.J. (2018). Robotic-assisted Laparoscopic Partial Cystectomies (RAPC) for Urachal Diseases—The Ideal Choice. Eur. Urol. Suppl..

[21] Chaabouni A., Samet A., Mseddi M.A., Rebai N., Harbi H., Hadjslimene M. (2021). Urachal mucinous cystadenoma: An exceptional entity. Urol. Case Rep..

[22] Page M.J., Mckenzie J.E., Bossuyt P.M., Boutron I., Hoffmann T.C., Mulrow C.D., Shamseer L., Tetzlaff J.M., Akl E.A., Brennan S.E. (2021). The PRISMA 2020 statement: An updated guideline for reporting systematic reviews. BMJ.

[23] Wells G.A., Shea B., O’connell D., Peterson J., Welch V., Losos M., Tugwell P. (2011). The Newcastle-Ottawa Scale (NOS) for Assessing the Quality of Nonrandomized Studies in Meta-Analyses.

[24] Moola S., Munn Z., Tufanaru C., Aromataris E., Sears K., Sfetcu R., Currie M., Lisy K., Qureshi R., Mattis P., Aromataris E., Munn Z. (2020). Chapter 7: Systematic reviews of etiology and risk. JBI Manual for Evidence Synthesis.

[25] Madeb R., Knopf J.K., Nicholson C., Donahue L.A., Adcock B., Dever D., Tan B.J., Valvo J.R., Eichel L. (2006). The use of robotically assisted surgery for treating urachal anomalies. BJU Int..

[26] Nayyar R., Anand A., Gupta N. (2009). VID-06.07: Robotic Partial Cystectomy for Urachal Adenocarcinoma: Results with Technique of Cystoscopically Optimized Surgical Margins. Urology.

[27] Correa J.J., Hakky T.S., Spiess P.E., Chuang T., Sexton W.J. (2010). Robotic-assisted partial cystectomy with en bloc excision of the urachus and the umbilicus for urachal adenocarcinoma. J. Robot. Surg..

[28] Kim D.K., Lee J.W., Park S.Y., Kim Y.T., Park H.Y., Lee T.Y. (2010). Initial experience with robotic-assisted laparoscopic partial cystectomy in urachal diseases. Korean J. Urol..

[29] Lee H.E., Jeong C.W., Ku J.H. (2010). Robot-assisted laparoscopic management of urachal cysts in adults. J. Robot. Surg..

[30] Tadtayev S., Bishop C.V., Adshead J.M. (2011). Robotic assisted partial cystectomy for urachal neoplasms: Experience at a regional uro-oncological centre. Eur. Urol. Suppl..

[31] Raynor M., Langston J., Selph P., Smith A., Nielsen M., Wallen E., Pruthi R. (2011). V1881 Initial Experience with Robotic-Assisted Approaches to Partial Cystectomy. J. Urol..

[32] Rivera M., Granberg C.F., Tollefson M.K. (2015). Robotic-assisted laparoscopic surgery of urachal anomalies: A single-center experience. J. Laparoendosc. Adv. Surg. Tech. A.

[33] James K., Vasdev N., Mohan S.G., Lane T., Adshead J.M. (2015). Robotic Partial Cystectomy for Primary Urachal Adenocarcinoma of the Urinary Bladder. Curr. Urol..

[34] Fode M., Pedersen G.L., Azawi N. (2016). Symptomatic urachal remnants: Case series with results of a robot-assisted laparoscopic approach with primary umbilicoplasty. Scand. J. Urol..

[35] Ahmed H., Howe A.S., Dyer L.L., Fine R.G., Gitlin J.S., Schlussel R.N., Zelkovic P.F., Palmer L.S. (2017). Robot-assisted Laparoscopic Urachal Excision in Children. Urology.

[36] Yong J., Law Z.W., Yang X.Y., Ng T.K., Yuen J.S.P. (2020). Robotic-assisted laparoscopic partial cystectomies (RAPC) for urachal diseases: Intuitive surgery for total umbilical tract excision and umbilectomy. Urol. Video J..

[37] Osumah T.S., Granberg C.F., Butaney M., Gearman D.J., Ahmed M., Gargollo P.C. (2021). Robot-Assisted Laparoscopic Urachal Excision Using Hidden Incision Endoscopic Surgery Technique in Pediatric Patients. J. Endourol..

[38] Stokkel L.E., van de Kamp M.W., Schaake E.E., Boellaard T.N., Hendricksen K., van Rhijn B.W., Mertens L.S. (2022). Robot-Assisted Partial Cystectomy versus Open Partial Cystectomy for Patients with Urachal Cancer. Urol. Int..

[39] Yamzon J., Kokorowski P., De Filippo R.E., Chang A.Y., Hardy B.E., Koh C.J. (2008). Pediatric robot-assisted laparoscopic excision of urachal cyst and bladder cuff. J. Endourol..

[40] Spiess P.E., Correa J.J. (2009). Robotic assisted laparoscopic partial cystectomy and urachal resection for urachal adenocarcinoma. Int. Braz. J. Urol..

[41] Allaparthi S., Ramanathan R., Balaji K.C. (2010). Robotic partial cystectomy for bladder cancer: A single-institutional pilot study. J. Endourol..

[42] Kosanovic R., Romero R.J., Arad J.K., Gallas M., Seetharamaiah R., Gonzalez A.M. (2014). Rare use of robotic surgery for removal of large urachal carcinoma. J. Robot. Surg..

[43] Aoun F., Peltier A., van Velthoven R. (2015). Bladder sparing robot-assisted laparoscopic en bloc resection of urachus and umbilicus for urachal adenocarcinoma. J. Robot. Surg..

[44] Williams C.R., Chavda K. (2015). En Bloc Robot-assisted Laparoscopic Partial Cystectomy, Urachal Resection, and Pelvic Lymphadenectomy for Urachal Adenocarcinoma. Rev. Urol..

[45] Shepler R., Zuckerman J.M., Troyer D., Malcolm J.B. (2016). Robotic-assisted laparoscopic partial cystectomy for symptomatic urachal hamartoma. Turk. J. Urol..

[46] Chen A., Chong J., Si Q., Haines K., Mehrazin R. (2018). Robotic approach to resection of villous adenoma of the urachus: A case report and literature review. J. Robot. Surg..

[47] Proskura A., Shpot E. (2018). Robot-assisted laparoscopic en-bloc partial cystectomy, urachal resection, umbilectomy and pelvic lymphadenectomy with intracorporeal ultrasonography for urachal adenocarcinoma: A case report. Eur. Urol. Suppl..

[48] George R., Swerdloff D., Akgul M., Nazeer T., Mian B.M. (2021). A rare case of urachal inflammatory myofibroblastic tumor. Urol. Case Rep..

[49] Connor J., Zheng Y., Stark A., Smith T., Grubb R.L. (2021). Umbilical sparing robotic partial cystectomy for localized urachal adenocarcinoma: A case report. Urol. Case Rep..

[50] Lough C.P., Rosen G.H., Murray K.S. (2021). Robotic excision of a calcified urachal cyst: A video case report. Urol. Video J..

[51] Zahr R.A., Colinet V., Mattlet A., Jabbour T., Diamand R. (2021). Robotic Partial Cystectomy for Urachal Carcinoma: A Case Report and Review of the Literature. Case Rep. Urol..

[52] Arena S., Rossanese M., Di Fabrizio D., Romeo C., Ficarra V., Impellizzeri P. (2021). Robot-assisted excision of urachal cyst: Case report in a child. Ann. Pediatr. Surg..

[53] Shin H.B., Park H.S., Kim J.H., Park J. (2021). A rare case of eosinophilic cystitis involving the inside and outside of the urinary bladder associated with an infected urachal cyst. BMC Urol..

[54] Park J.J., Bin Kim W., Lee K.W., Kim J.M., Kim Y.H., Kim J.H., Moon A., Kim S.H., Lee S.W. (2021). Robot-assisted laparoscopic intracorporeal urachal mass resection and partial cystectomy for a huge urachal adenocarcinoma: A case report and review of literature. J. Men’s Health.

[55] Kochvar A.P., Bednar G., Albani J.M. (2023). Low-Grade Urachal Cystadenoma With Abundant Calcification Removed Using Robot-Assisted Laparoscopy: A Case Report. Cureus.

[56] Bogaerts Q., Vanthoor J., Goethuys H., Raskin Y. (2023). Cystoscopic and robotic-assisted laparoscopic excision of a rare urachus neoplasm by partial cystectomy. Urol. Video J..

[57] Kunitsky K.D., Almajedi M., Snajdar E., Adams P., Nelson R. (2024). Single-Port Robotic-Assisted Excision of the Urachal Remnant in an Adult Female: A Case Report. Cureus.

[58] Hemal S., Sobhani S., Hakimi K., Rosenberg S., Gill I. (2024). Single-Port Robot assisted partial cystectomy for urachal adenocarcinoma. Int. Braz. J. Urol..

[59] Hamasaki S., Kaneko G., Yabuno A., Miyama Y., Hiruta S., Hagiwara M., Shirotake S., Yasuda M., Oyama M. (2024). Robot-Assisted Partial Cystectomy Using the “Double Bipolar Method”. Cureus.

[60] Elsheikh M., Oxley J., Qureshi F., Thornton M. (2024). A Rare Case of Urinary Bladder Leiomyoma Invading Urachal Remnant Managed With Robotic Partial Cystectomy. Cureus.

[61] Thiesfeldt D.L., Seth A., Ellsworth P. (2024). A large urachal cyst presenting with lower urinary tract symptoms and a falsely elevated post-void residual on bladder scan. Urol. Case Rep..

[62] Rich J.M., Gonzalez A.N., Murray K.S. (2024). Robotic urachal cyst removal: Video case report and tutorial for robotic surgical trainees. Urol. Video J..

[63] Checcucci E., Amparore D., Fiori C., Manfredi M., Ivano M., Di Dio M., Niculescu G., Piramide F., Cattaneo G., Piazzolla P. (2020). 3D imaging applications for robotic urologic surgery: An ESUT YAUWP review. World J. Urol..

[64] Tanaka K., Misawa T., Baba Y., Ohashi S., Suwa K., Ashizuka S., Yoshizawa J., Ohki T. (2019). Surgical Management of Urachal Remnants in Children: Open versus Laparoscopic Approach. Medicine.

[65] Bada M., De Concilio B., Crocetto F., Creta M., Silvestri T., Di Mauro M., Celia A. (2020). Laparoscopic Radical Cystectomy with Extracorporeal Urinary Diversion: An Italian Single-Center Experience with 10-Year Outcomes. Minerva Urol. Nefrol..

[66] Wang B., Li X., Ming S., Ma X., Li H., Ai Q., Zhang X. (2016). Combined Extraperitoneal and Transperitoneal Laparoscopic Extended Partial Cystectomy for the Treatment of Urachal Carcinoma. J. Endourol..

[67] Sui Y., Zhang Z., Zhao K., Zhang Y., Wang Z., Zhu G., Yang H., Li X., Wang Q., Yin X. (2023). A Novel Extraperitoneal Approach Exploration for the Treatment of Urachal Mass: A Retrospective Observational Single-Center Study. J. Chin. Med. Assoc..

[68] Ryan P.C., Kelly C., Afridi I., Fawaz A., Aboelmagd M., Cullen I.M., Keane J.P., Daly P.J. (2023). Surgical Treatment of Urachal Remnants in an Adult Population—A Single-Centre Experience. Ir. J. Med. Sci..

[69] Mylonas K.S., O’Malley P., Ziogas I.A., El-Kabab L., Nasioudis D. (2017). Malignant urachal neoplasms: A population-based study and systematic review of literature. Urol. Oncol..

[70] Aylward P., Samson K., Raynor S., Cusick R. (2020). Operative Management of Urachal Remnants: An NSQIP-Based Study of Postoperative Complications. J. Pediatr. Surg..

[71] Mirto B.F., Barone B., Balsamo R., Abate M., Caputo V.F., Sciarra A., Calogero A., Romano L., Napolitano L., Sciorio C. (2024). Early and Late Post-Procedural Complications in Different Orthotopic Neobladder Surgical Approaches: A Systematic Review. Surg. Oncol..

[72] Wang J., Hu K., Wang Y., Wu Y., Bao E., Wang J., Tan C., Tang T. (2023). Robot-Assisted versus Open Radical Prostatectomy: A Systematic Review and Meta-Analysis of Prospective Studies. J. Robot. Surg..

[73] Cella L., Basile G., Moretto S., Paciotti M., Hurle R., Lughezzani G., Avolio P.P., Piccolini A., Mancon S., Lazzeri M. (2024). Robotic Assisted vs. Open Radical Cystectomy: An Updated Systematic Review and Meta-Analysis. J. Robot. Surg..

[74] Ge S., Zeng Z., Li Y., Gan L., Meng C., Li K., Wang Z., Zheng L. (2023). The Role of Robotic Retroperitoneal Lymph Node Dissection in Testicular Cancer: A Systematic Review and Meta-Analysis. Int. J. Surg..

[75] Patel K.N., Salunke A., Bakshi G., Jayaprakash D., Pandya S.J. (2022). Robotic-Assisted Video-Endoscopic Inguinal Lymphadenectomy (RAVEIL) and Video-Endoscopic Inguinal Lymphadenectomy (VEIL) versus Open Inguinal Lymph-Node Dissection (OILND) in Carcinoma of Penis: Comparison of Perioperative Outcomes, Complications and Oncological Outcomes. A Systematic Review and Meta-Analysis. Urol. Oncol..

[76] Veccia A., Antonelli A., Francavilla S., Simeone C., Guruli G., Zargar H., Perdoná S., Ferro M., Carrieri G., Hampton L.J. (2020). Robotic versus Other Nephroureterectomy Techniques: A Systematic Review and Meta-Analysis of Over 87,000 Cases. World J. Urol..

[77] Baghli A., Achit H., Audigé V., Larré S., Branchu B., Balkau B., Eschwege P., Hubert J., Mazeaud C. (2023). Cost-Effectiveness of Robotic-Assisted Surgery vs. Open Surgery in the Context of Partial Nephrectomy for Small Kidney Tumors. J. Robot. Surg..

[78] Calpin G.G., Ryan F.R., McHugh F.T., McGuire B.B. (2023). Comparing the Outcomes of Open, Laparoscopic and Robot-Assisted Partial Nephrectomy: A Network Meta-Analysis. BJU Int..

[79] Mehta A., Ng J.C., Awuah W.A., Huang H., Kalmanovich J., Agrawal A., Abdul-Rahman T., Hasan M.M., Sikora V., Isik A. (2022). Embracing Robotic Surgery in Low- and Middle-Income Countries: Potential Benefits, Challenges, and Scope in the Future. Ann. Med. Surg..

[80] Wang L., Zeng W., Wu Y., Gong Z. (2024). Comparison of Clinical Efficacy and Safety Between Robotic-Assisted and Laparoscopic Adrenalectomy for Pheochromocytoma: A Systematic Review and Meta-Analysis. J. Robot. Surg..

[81] Lawrie L., Gillies K., Duncan E., Davies L., Beard D., Campbell M.K. (2022). Barriers and Enablers to the Effective Implementation of Robotic Assisted Surgery. PLoS ONE.

[82] Gallagher A.G., De Groote R., Paciotti M., Mottrie A. (2022). Proficiency-based Progression Training: A Scientific Approach to Learning Surgical Skills. Eur. Urol..

[83] Dell’oglio P., Turri F., Larcher A., D’hondt F., Sanchez-Salas R., Bochner B., Palou J., Weston R., Hosseini A., Canda A.E. (2022). Definition of a Structured Training Curriculum for Robot-assisted Radical Cystectomy with Intracorporeal Ileal Conduit in Male Patients: A Delphi Consensus Study Led by the ERUS Educational Board. Eur. Urol. Focus.

[84] Diamand R., D’Hondt F., Mjaess G., Jabbour T., Dell’Oglio P., Larcher A., Moschini M., Quackels T., Peltier A., Assenmacher G. (2023). Teaching robotic cystectomy: Prospective pilot clinical validation of the ERUS training curriculum. BJU Int..

[85] Larcher A., De Naeyer G., Turri F., Dell’oglio P., Capitanio U., Collins J.W., Wiklund P., Van Der Poel H., Montorsi F., Mottrie A. (2019). The ERUS Curriculum for Robot-assisted Partial Nephrectomy: Structure Definition and Pilot Clinical Validation. Eur. Urol..

[86] Pecoraro A., Territo A., Boissier R., Hevia V., Prudhomme T., Piana A., Marco B.B., Gallagher A.G., Serni S., Decaestecker K. (2024). Proposal of a standardized training curriculum for open and robot-assisted kidney transplantation. Minerva Urol. Nephrol..

[87] Mottrie A., Novara G., van der Poel H., Dasgupta P., Montorsi F., Gandaglia G. (2016). The European Association of Urology Robotic Training Curriculum: An Update. Eur. Urol. Focus.

[88] Thornblade L.W., Fong Y. (2021). Simulation-Based Training in Robotic Surgery: Contemporary and Future Methods. J. Laparoendosc. Adv. Surg. Tech. A.

[89] Bergholz M., Ferle M., Weber B.M. (2023). The benefits of haptic feedback in robot assisted surgery and their moderators: A meta-analysis. Sci. Rep..

[90] Sanchez A., Herrera L., Teixeira A., Cheatham M., Gibson D., Lam V., Guevara O. (2023). Improving efficiency and reducing costs in robotic surgery: A Lean Six Sigma approach to optimize turnover time. J. Robot. Surg..

[91] Nayeemuddin M., Daley S.C., Ellsworth P. (2013). Modifiable factors to decrease the cost of robotic-assisted procedures. AORN J..

[92] Basile G., Gallioli A., Diana P., Gallagher A., Larcher A., Graefen M., Harke N., Traxer O., Tilki D., Van Der Poel H. (2024). Current Standards for Training in Robot-assisted Surgery and Endourology: A Systematic Review. Eur. Urol..

[93] Etta P., Chien M., Wang Y., Patel A. (2024). Robotic partial nephrectomy: Indications, patient selection, and setup for success. Urol. Oncol..

[94] Balbay M.D., Koc E., Canda A.E. (2017). Robot-assisted radical cystectomy: Patient selection and special considerations. Robot. Surg..

[95] Palagonia E., Mazzone E., De Naeyer G., D’hondt F., Collins J., Wisz P., Van Leeuwen F.W.B., Van Der Poel H., Schatteman P., Mottrie A. (2020). The safety of urologic robotic surgery depends on the skills of the surgeon. World J. Urol..

[96] Leddy L., Lendvay T., Satava R. (2010). Robotic surgery: Applications and cost effectiveness. Open Access Surg..

[97] Maccraith E., Forde J.C., Davis N.F. (2019). Robotic simulation training for urological trainees: A comprehensive review on cost, merits and challenges. J. Robot. Surg..

[98] Knudsen J.E., Ghaffar U., Ma R., Hung A.J. (2024). Clinical applications of artificial intelligence in robotic surgery. J. Robot. Surg..

[99] Guni A., Varma P., Zhang J., Fehervari M., Ashrafian H. (2024). Artificial Intelligence in Surgery: The Future is Now. Eur. Surg. Res..

[100] Iftikhar M., Saqib M., Zareen M., Mumtaz H. (2024). Artificial intelligence: Revolutionizing robotic surgery: Review. Ann. Med. Surg..

